# Knowledge, Attitudes, and Practices Toward the Prevention of COVID-19 in Bangladesh: A Systematic Review and Meta-Analysis

**DOI:** 10.3389/fmed.2022.856156

**Published:** 2022-06-06

**Authors:** Ahsan Raquib, Radwan Raquib, Safayet Jamil, Ahmed Hossain, Firoj al-Mamun, Mohammed A. Mamun

**Affiliations:** ^1^CHINTA Research Bangladesh, Dhaka, Bangladesh; ^2^Faculty of Veterinary, Animal and Biomedical Sciences, Sylhet Agricultural University, Sylhet, Bangladesh; ^3^Department of Pharmacy, Khwaja Yunus Ali University, Sirajgonj, Bangladesh; ^4^Department of Public Health, North South University, Dhaka, Bangladesh; ^5^Global Health Institute, North South University, Dhaka, Bangladesh; ^6^Department of Public Health and Informatics, Jahangirnagar University, Dhaka, Bangladesh; ^7^Department of Public Health, University of South Asia, Dhaka, Bangladesh; ^8^Department of Public Health, Daffodil International University, Dhaka, Bangladesh

**Keywords:** COVID-19 knowledge, COVID-19 preventive behaviors, knowledge, attitudes, practice (KAP), pandemic, systematic review and meta-analysis

## Abstract

**Background:**

Numerous studies on knowledge, attitude, and practice (KAP) about the prevention of COVID-19 infections are available in Bangladeshi contexts, with results that vary significantly. However, no earlier attempt has been made to analyze the available COVID-19 KAP studies in Bangladesh, which is incorporated in this meta-analysis for the first time.

**Methods:**

Following the PRISMA guidelines, articles relevant to COVID-19 KAP that were conducted among the Bangladeshi population were found in databases such as PubMed, Scopus, CINAHL, Google Scholar, and ResearchGate. Random-effect meta-analysis was used to generate a pooled prevalence of knowledge, attitude, and practice level toward the prevention of COVID-19 infection.

**Results:**

This review included 18 articles that were published between March 2020 and November 2021. Overall, 89.87% (95% CI: 67.71–97.40) understood about COVID-19 symptoms, 92.09% (95% CI: 84.32–96.18) knew about how it spreads, and 79.51% (95% CI: 59.38–91.15) knew about how to treat it. The public's perception of controlling COVID-19 is mixed, with only 44.16% (95% CI: 35.74–52.93) and 60.28% (95% CI: 49.22–70.38) believing the country would win the struggle against the pandemic and the infection will be successfully controlled, respectively. Although overall COVID-19 preventative practice was good, subgroup analysis found that men had a poor practice toward controlling the infection. The practice of avoiding crowded places (70.15%) and maintaining social distance (77.17%) was found to be satisfactory in institution-based studies.

**Conclusion:**

The findings of this study revealed that the Bangladeshi population had a good awareness of COVID-19 symptoms, treatment, attitudes, and behaviors. The findings of this study are likely to aid Bangladeshi governments and policymakers in putting evidence into action by identifying gaps and emphasizing the importance of educating the less informed public about COVID-19 transmission.

## Introduction

The COVID-19 pandemic caused by the SARS-CoV-2 virus has become the most public health concern affecting all aspects of life (1). The first case of COVID-19 was diagnosed in Wuhan, China, in late December 2019 (2, 3), by a patient with unexplained pneumonia etiology (4). However, as of November 22, 2021, approximately 258 million people are reported to be infected with the virus globally, whereas 5.15 million lost their lives. The World Health Organization declared it a pandemic due to its devastating effects on all aspects of health and the quality of life (5). The first COVID-19 case diagnosis in Bangladesh was reported on March 8, 2020 (6, 7). The country has been alleged to have poor healthcare facilities and skilled manpower to tackle any health emergencies. This situation worsened during the pandemic due to the overwhelming number of cases (8). As of November 22, 2021, 1.57 million Bangladeshi people have been tested positive for the virus, and the number of mortalities is 28,000. However, to mitigate the virus's rapid transmission, approaches to medication or therapeutic such as social movement restriction, lockdown, and quarantine have been imposed (9).

The impact of COVID-19 is severe among these people with chronic medical conditions such as diabetes, cancer, heart disease, and circulatory disorders, although general people are not escaping from the stressful situation created by the pandemic (10). Consequently, unwanted fear, panic, and worry related to being infected with the virus, loss of beloved ones, and economic crisis occur, whereas people have been reported at a higher risk of issues such as common mental health problems (i.e., depression, anxiety, and stress) and poor physical health along with inflammatory diseases (5, 11). Nearly half of the Bangladeshi people have been reported suffering from mental health problems (higher than the prevalence rates of mental disorders during the normal period), as estimated by a recent meta-analysis of the studies conducted during the COVID-19 pandemic (12). However, Bangladesh, like other countries, has implemented various safety precautions to mitigate the transmission, including pedestrianizing flow, confining them at home, allowing them to work from home, increasing awareness, disseminating information, closing schools, and providing other public assistance (13, 14).

It is said that public response to a disease is determined by knowledge and understanding of its etiology, signs and symptoms, treatment, and even prevention, which are expressed by their attitudes and practices toward the diseases (15, 16). The risk of disease-related adverse outcomes in a population increases if negative attitudes and practices are not possibly measured for modification. Therefore, assessing public understanding, perception, and experience related to COVID-19 is essential to visualize their preparedness for the pandemic (15, 16). This helps government and health authorities determine how to adopt the programs to control the outbreak. Therefore, many studies have been conducted to assess Bangladesh's knowledge, attitudes, and practice (KAP) toward COVID-19, but there is a lack of evidence generated from a systematic evaluation.

Given the nature of the COVID-19 pandemic, regular update of scientific literature is essential for initiating empirical evidence on interventions and strategies to tackle the COVID-19 pandemic more conveniently. For this purpose, a comprehensive and timely updated systematic evaluation of existing evidence is highly needed, whereas a few systematic review and meta-analysis has been published on the KAP of COVID-19 (15, 17–19). Notwithstanding, region- or country-based systematic reviews lack; for instance, Ethiopia (17, 20) and the United States (20) have reported publishing country-based systematic reviews on the KAP of COVID-19. Furthermore, knowledge and public perception being culture-based, interventions should focus on the data from the respective culture. Despite the global evidence, it is hard to achieve any policy directions and implement them due to cultural sensitivity. As a result, in order to better grasp the KAP for COVID-19 prevention in Bangladesh, a systematic review and meta-analysis is undertaken herein to better comprehend the infection's control.

## Methods

### Search Strategy

Preferred Reporting Items for Systematic Reviews and Meta-Analyses (PRISMA) protocol was used as a guideline for performing the present systematic review. To identify relevant studies for including in this review, a systematic literature search was conducted on the relevant databases like PubMed, Scopus, CINAHL, and Google Scholar between July 1, 2021, and July 10, 2021. In addition, random searches were done in ResearchGate to include missing literature. The search strategy included keywords in the combination of (i) knowledge OR attitude OR perception OR practice, (ii) corona virus OR novel coronavirus OR COVID-19 OR severe acute respiratory syndrome (iii) Bangladesh.

### Eligibility Criteria

For inclusion of the literature, the following criteria were applied to the retrieved studies (i) being a cross-sectional observation study, (ii) having full-text access, (iii) being concerned with any of the KAP of COVID-19 related questions (Supplementary File), (iv) being published in a peer-reviewed journal in English, (iv) being conducted between March 2020 and November 2021, and (v) being conducted among Bangladeshi residents. In addition, literature like editorial, letter to the editor, commentary, perspective, preprint, and articles that failed to fulfill the aforementioned eligibility criteria were eliminated from this study.

### Study Selection

**Figure 1:**
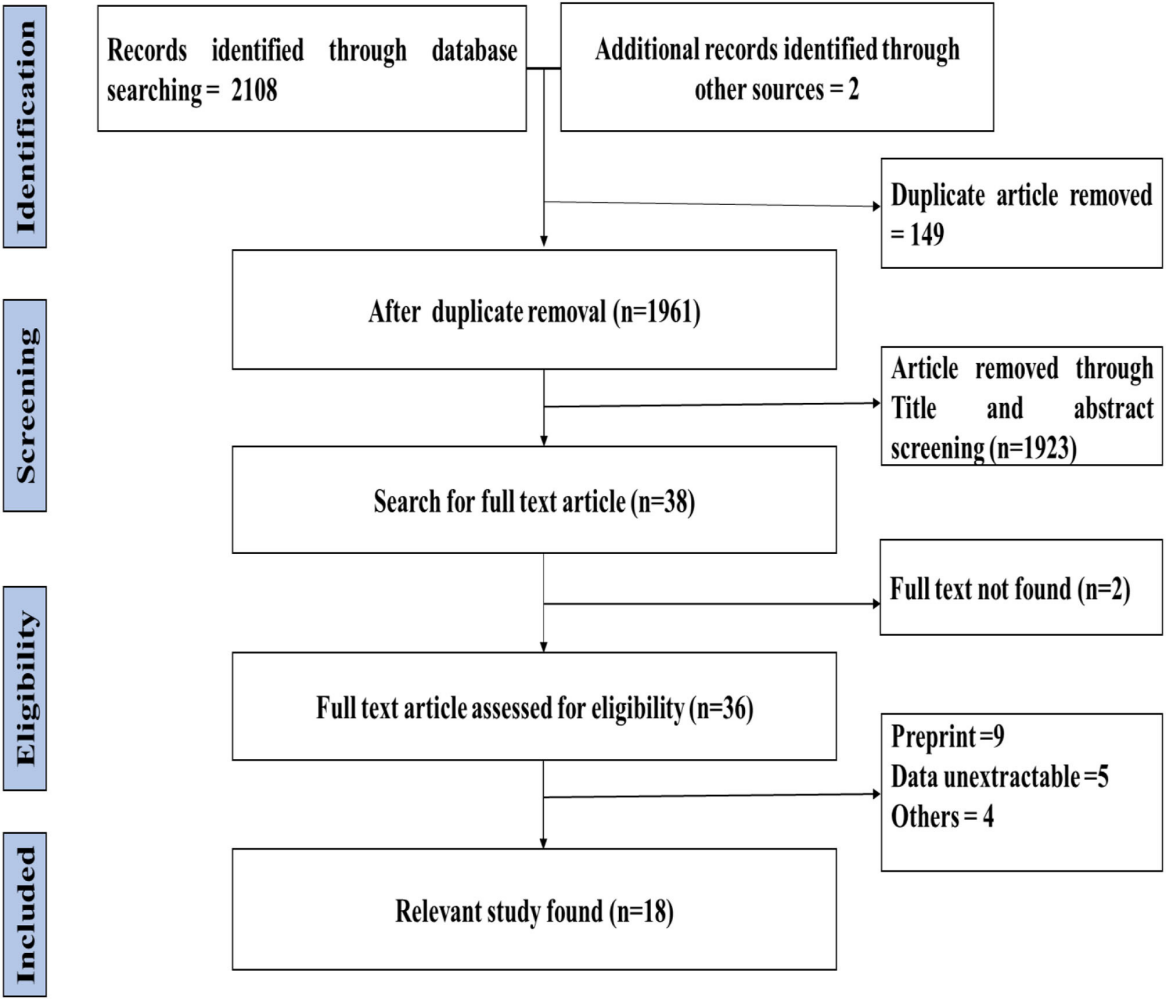
PRISMA flow diagram of literature search and study selection.

To remove duplicate studies, articles (*N* = 2,110) retrieved from the database searches were exported to reference manager Zotero and Excel 2013. After carefully removing duplicate articles (*n* = 149), the title and abstracts of the remaining 1,923 studies were screened for pertinent studies, followed by a freely accessible study selection and relevant study selection based on eligibility criteria. Through title and abstract screening, 36 articles were left for full-text reading, and based on the eligible criteria, 18 articles were finally selected to be included in this review (Figure 1).

### Quality Assessment

Risk of Bias Tool (RoBT) that was developed by Hoy et al. (21) was used to assess the risk of bias of all the included studies in this review. This assessment evaluated 10 items in which external validity (items one to four) and internal validity (items five to ten) were measured (Supplementary File). Each item of RoBT is scored “0” (risk of bias absent) or “1” (risk of bias present), where 10 is the highest score reflecting a greater risk of bias. However, based on the RoBT score, studies were classified as low risk (0–3), moderate risk (4–6), and high risk (7–10).

### Data Extraction

From the eligible studies, the following information was retrieved in an excel file: first author, sample collection date, publication year, participant's characteristics (age, sex, education level, and occupation), focus group, study design, number of participants, and percentage of positive answers on each selected KAP (knowledge, attitude, and practice) questions.

### Data Analysis

R studio version 4.1.0 was used to perform a meta-analysis of the data exported from an Excel spreadsheet. I^2^ (%) statistics were applied for the evaluation of study heterogeneity, where 25, 50, and 75% represented low, moderate, and severe heterogeneity, respectively. A random-effect model was employed to conduct the meta-analysis because of high heterogeneity, and the results were presented in forest plots. Additional subgroup analysis was conducted for study design and gender. Publication bias assessment was visualized by funnel plot and for rigorous assumptions egger's regression test was performed.

## Results

### Characteristics of the Included Studies

#### General Description of the Included Studies

All the selected studies (*n* = 18) comprised a total of 16,443 participants, where 8,523, 7,885, and 15 participants were male, female, and others, respectively, with a sample size range from 110 to 2,157. Twelve studies (6, 8, 9, 22–30) employed a community-based cross-sectional study design, and the other six (1, 2, 31–34) used an institutional-based cross-sectional study design. Among the included studies, 11 were published in 2020, and seven were published in 2021 (Table 1).

**Table 1:** Demographic characteristics of the participants.

**References**	**Study design**	**Participants**	**Mean age of participants**	**Sampling date**	**Focus group**	**Risk of Bias**	**Quality score**	**Questionnaire administration**
Rahman et al. (22)	CBCS	616 (male: 58.44%)	Not mentioned	Mid of May to end of May 2020	General people	Low	2	SAOB
Hossain et al. (31)	IBCS	378 (male: 32.54%)	17.03 (±0.17)	August 7 to August 18 2020	Students	Low	3	SAOB
Pervez et al. (23)	CBCS	315 (male: 54.92%)	26.54 (± 3.05)	May 1 to May 25, 2020	Urban people	Low	3	SAOB
Ahmed et al. (32)	IBCS	200 (male: 66%)	22 (± 2.09)	July 3 2020 to July 15 2020	Public university students	Low	3	SAOB
Roy et al. (33)	IBCS	110 (male: 77.27%)	Not mentioned	June 2020	Sub Assistant Agriculture Officers	Moderate	4	Face-to-face interviews
Akram et al. (34)	IBCS	139 (male: 75.54%)	30.1 (±6.1)	April and May 2020	Healthcare workers	Low	3	Not mentioned
Rahman et al. (2)	IBCS	952 (male: 49.58%)	15–30	Not mentioned	Public university students	Low	2	SAOB
Hossain et al. (24)	CBCS	1,861 (male: 64.54%, third gender: 0.81%)	Not mentioned	March 19 to April 15, 2020	General people	Low	3	Not mentioned
Rahman et al. (25)	CBCS	1,520 (male: 62.17%)	30.1 (± 6.1)	March 15 to April 15,2020	General people	Low	2	Online based
Islam et al. (26)	CBCS	406 (male: 53.20%)	44.9 (±12.1)	August and September 2020	Slum dwellers	Low	3	Face to face interview
Anwar et al. (9)	CBCS	1,869 (male: 00%)	29.55 (±12.01)	Not mentioned	Adult women	Low	3	Telephone, online, or in-person interviews
Hossain et al. (27)	CBCS	1,056 (male: 63.26%)	31.6 (±10.56)	May 10 to May 16 May 2020	Adult population	Low	1	SAOB
Paul et al. (28)	CBCS	1,589 (male: 60.48%)	Not mentioned	March 22 to March 28, 2020	General people	Low	2	SAOB
Hossain et al. (8)	CBCS	2,157 (male: 54.06%)	13–90	April 4 to May 2, 2020	General people	Low	2	SAOB
Ahmed et al. (29)	CBCS	1,222 (male: 61.37%)	30.77 (±12.1)	June 27 to July 20, 2020	General people	Moderate	4	Face to face interviews and Online-based
Ferdous et al. (6)	CBCS	2,017 (male: 59.79%)	12–64	March 29 to April 20, 2020	General people	Low	2	SAOB
Wadood et al. (1)	IBCS	305 (male: 74.17%)	20.66 (±1.78)	March 11 to March 19, 2020	Students	Moderate	4	Face to face interview
Ahmad et al. (30)	CBCS	517 (male: 36.94%)	Not mentioned	April 15 to April 30, 2020	Medical students and their family members	Low	3	Online based

*IBCS, Institutional-based cross-sectional study; CBCS, Community-based cross-sectional study; SAOB, Self-administered online-based*.

#### Measurement Used in the Included Studies

Knowledge of COVID-19 was assessed by the domains of (i) symptoms (fever, dry cough, respiratory signs, weakness, diarrhea, headache, and sore throat), (ii) transmission (spread through respiratory droplet), and (iii) treatment (no specific treatment is available), whereas those items were responded using “Yes/No/Don't know,” or “True/False/Not sure” scheme in the included studies. In case of practices related to COVID-19 (wash hands regularly, maintain social distance, avoid the crowded place, and always wear a mask when going outside) and attitudes concerning COVID-19 (COVID-19 pandemic will be successfully controlled, and Bangladesh can win the battle against the COVID-19 pandemic), the included studies collected response using “Yes/No/Don't know” or “Strongly agree/Agree/Neutral/Disagree/Strongly disagree” items. The responses of “Yes,” “True,” or “Strongly agree/Agree” are considered positive responses for analysis herein.

### Knowledge About COVID-19 Symptoms

**Figure 2:**
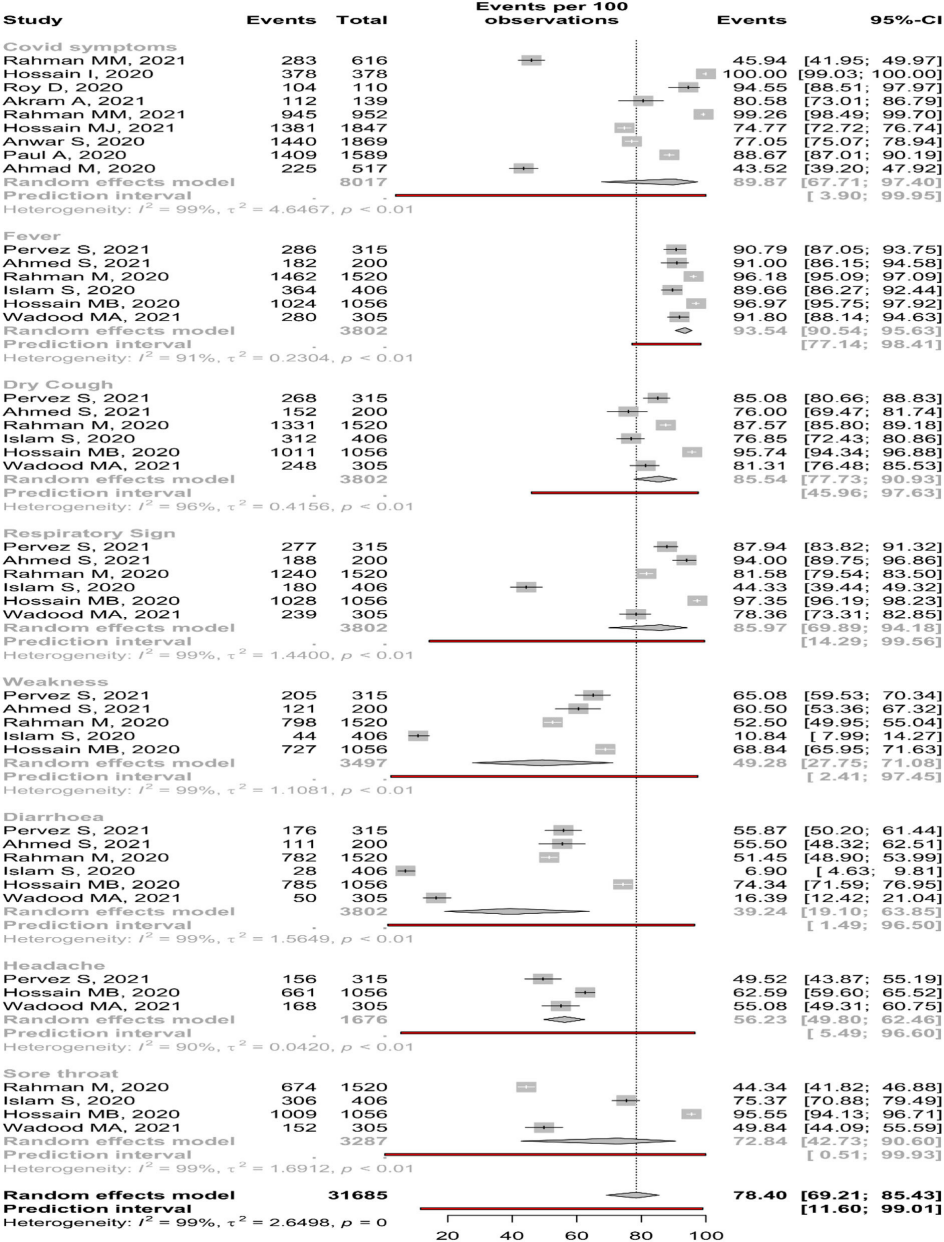
Knowledge about COVID-19 symptoms among Bangladeshi residents.

Nine studies reported about overall knowledge of participants about COVID-19 symptoms, where 89.87% (95%, CI: 67.71–97.40%) participants had positive knowledge. In addition, six studies reported knowledge of fever, dry cough, and respiratory signs, where 93.54, 85.54, and 85.97% positive knowledge was encountered. Conversely, the lowest percentage of participants' knowledge was on diarrhea (39.24%, 95% CI: 19.10–63.85%) (Table 2). The forest is presented in Figure 2.

**Table 2:** Pooled prevalence of knowledge about COVID-19 among Bangladeshi residents.

**Knowledge about COVID-19**		**No of study**	**Sample size**	**Percentage, (%)**	**95% CI**	**I^**2**^ (%)**	***p*** **-Value**	**Eggers test**
**COVID-19 symptoms**								
Fever		6	3,802	93.54	90.54–95.63	90.6	<0.01	0.30
	Male	3	521	89.64	86.71–91.98	15.7	0.31	
	Female	3	400	91.93	86.52–95.29	65.8	0.05	
Dry cough		6	3,802	85.54	77.73–90.93	96.0	<0.01	0.86
	Male	3	521	82.05	77.37–85.94	61.3	0.08	
	Female	3	400	77.14	68.48–83.99	79.6	<0.01	
Respiratory sign		6	3,802	85.97	69.89–94.18	98.8	<0.01	0.42
	Male	3	521	79.36	55.29–92.28	97.3	<0.01	
	Female	3	400	88.61	42.69–98.78	97.8	<0.01	
Weakness		5	3,497	49.28	27.75–71.08	98.7	<0.01	0.56
	Male	3	521	46.12	20.47–74.00	98.1	<0.01	
	Female	3	400	33.14	7.24–75.89	97.7	<0.01	
Diarrhea		6	3,802	39.24	19.10–63.85	99.0	<0.01	0.23
	Male	3	521	37.24	13.60–69.12	97.9	<0.01	
	Female	3	400	26.26	4.98–70.77	97.0	<0.01	
Headache		3	1,676	56.23	49.80–62.46	89.5	<0.01	0.23
Sore throat		4	3,287	72.84	42.73–90.60	99.4	<0.01	0.17
Overall symptoms		9	8,017	89.87	67.71–97.40	98.9	<0.01	0.50
**COVID-19 transmission**								
Spread through respiratory droplet		10	8,533	92.09	84.32–96.18	99.1	<0.01	<0.01
	Male	3	1554	85.67	65.11–95.04	95.6	<0.01	
	Female	3	1,069	85.69	61.45–95.74	94.7	<0.01	
**COVID-19 treatment**								
No-specific treatment available		11	11,634	79.51	59.38–91.15	98.1	<0.01	0.41
	Male	1	1,206	80.18	77.84–82.34			
	Female	2	2,624	82.36	80.85–83.77	0.00	0.38	

The subgroup meta-analysis of COVID-19 symptoms reveals that participants of the community-based studies had higher knowledge about fever, dry cough, diarrhea, headache, and sore throat than institution-based studies. In the case of gender, the male had higher knowledge of fever, dry cough, weakness, and diarrhea (Supplementary Material).

### Knowledge About COVID-19 Transmission

**Figure 3:**
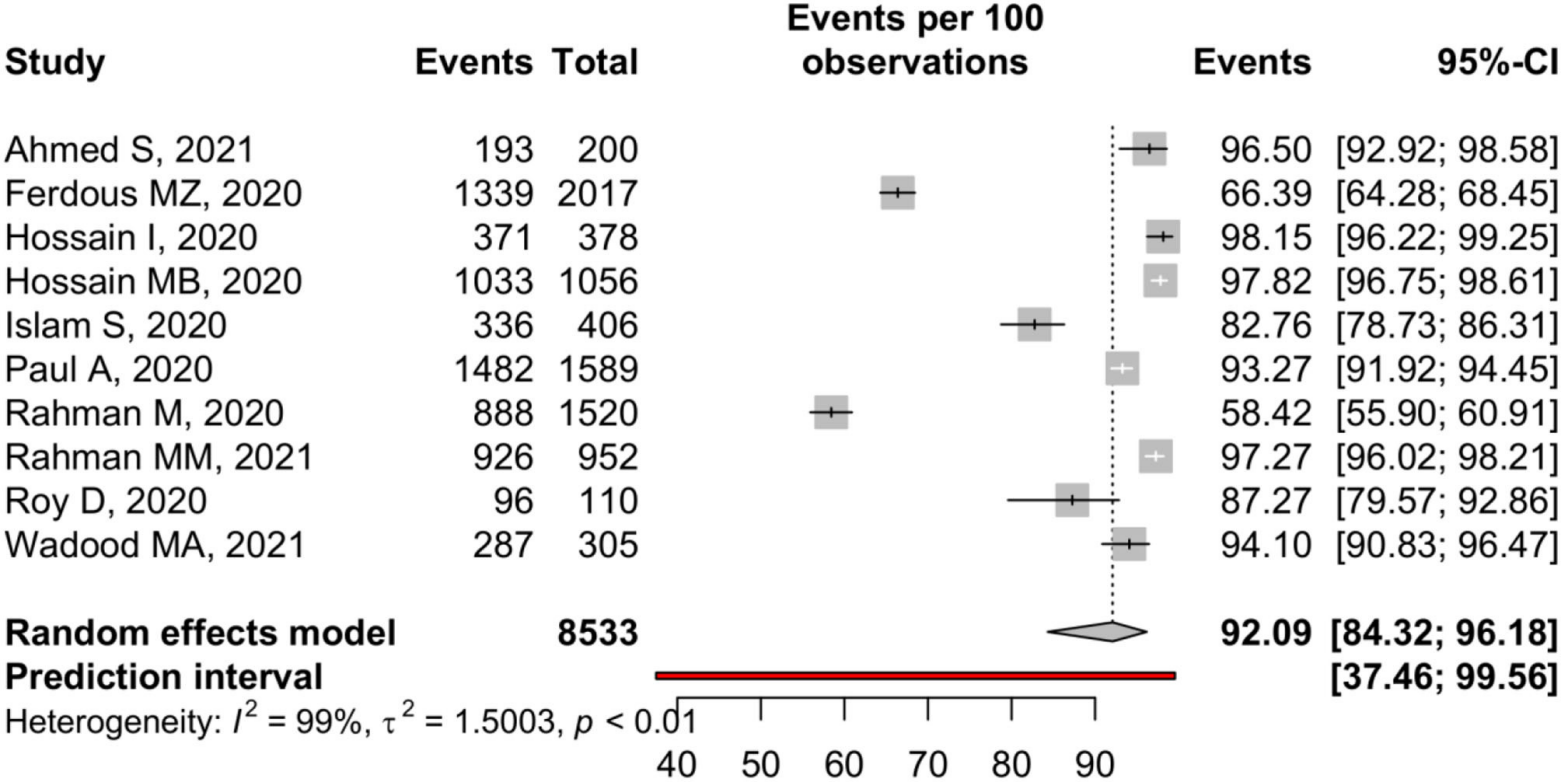
Knowledge about COVID-19 transmission among Bangladeshi residents.

Only 10 studies reported transmission, and random-effect meta-analysis estimated 92.09% (95% CI: 84.32–96.18%) participants were aware of the fact that COVID-19 can transmit through respiratory droplets. A substantial amount of study heterogeneity was identified (I^2^ = 99.1%), where Eggers tests showed a small-study effect (Table 2). The forest is presented in Figure 3.

In the subgroup analysis of gender, it was found that males (85.67%, 95% CI: 65.11–95.04%) and females (85.69%, 95% CI: 61.45–95.74%) had the almost same level of knowledge of COVID-19 transmission. Appropriate knowledge of transmission was estimated as 85.82 % (95%, CI: 66.41–94.88%) in CBCS and 95.82% (95%, CI: 92.50–97.70%) in IBCS (Supplementary Material).

### Knowledge About COVID-19 Treatment

**Figure 4:**
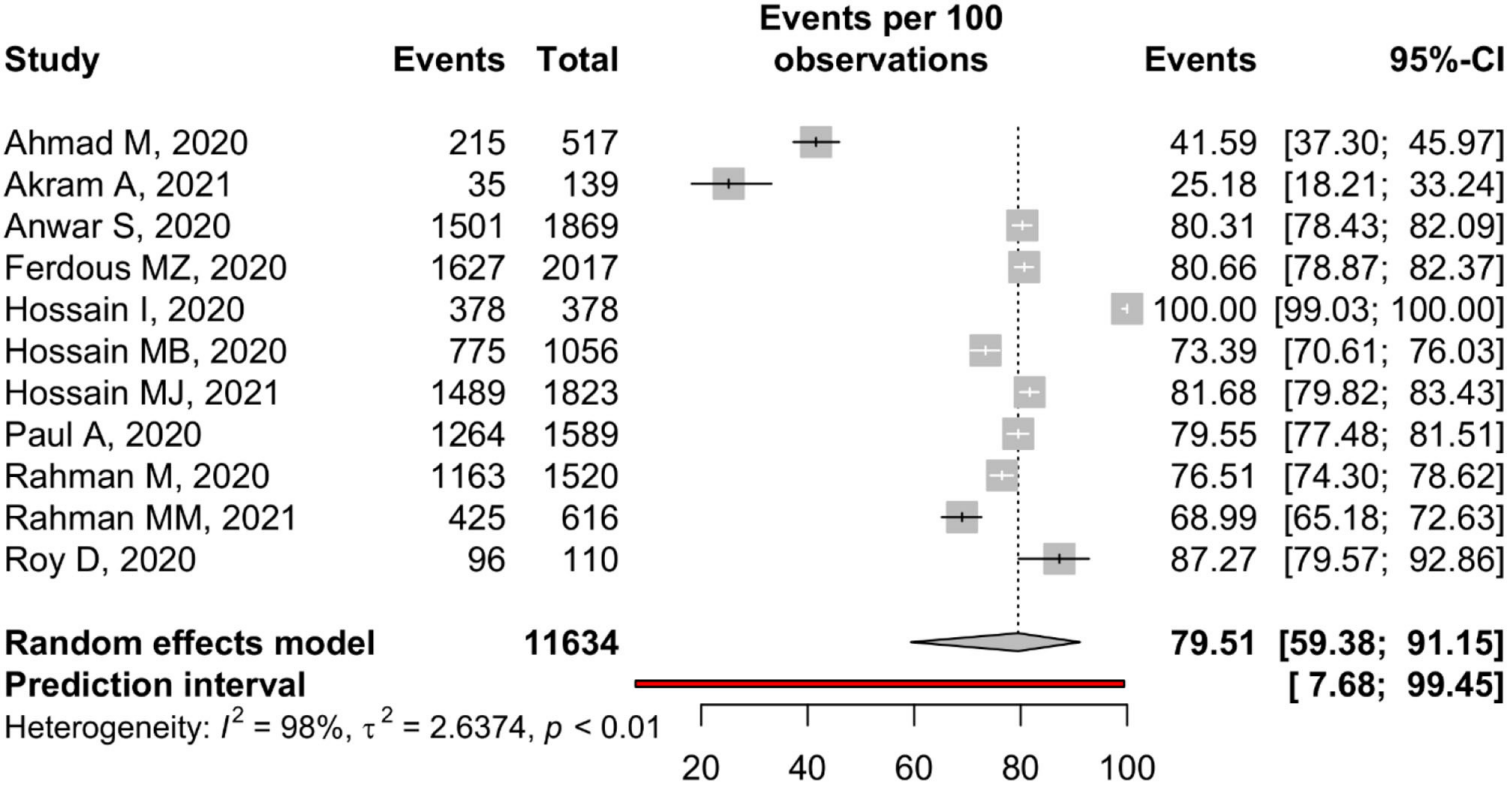
Knowledge about COVID-19 treatments among Bangladeshi residents.

Eleven studies reported the treatment of COVID-19, and 79.51% (95%, CI: 59.38–91.15%) of participants know that COVID-19 has no specific treatment. A substantial amount of study heterogeneity was identified (I^2^ = 98.1%), whereas a small-study effect was absent based on the Eggers test (*p* = 0.41) (Table 2). The forest is presented in Figure 4.

Participants of IBCS (95.84%, 95% CI: 11.58–99.98%) had better knowledge of COVID-19 treatment than the participants of CBCS (74.01%, 95% CI: 65.83–80.80%). Higher percentage of female (82.36%, 95% CI: 80.85–83.77%) knew about the unavailability of specific treatment of COVID-19 than male (80.18%, 95% CI: 77.84–82.34%) (Supplementary Material).

### Attitude Toward COVID-19

**Figure 5:**
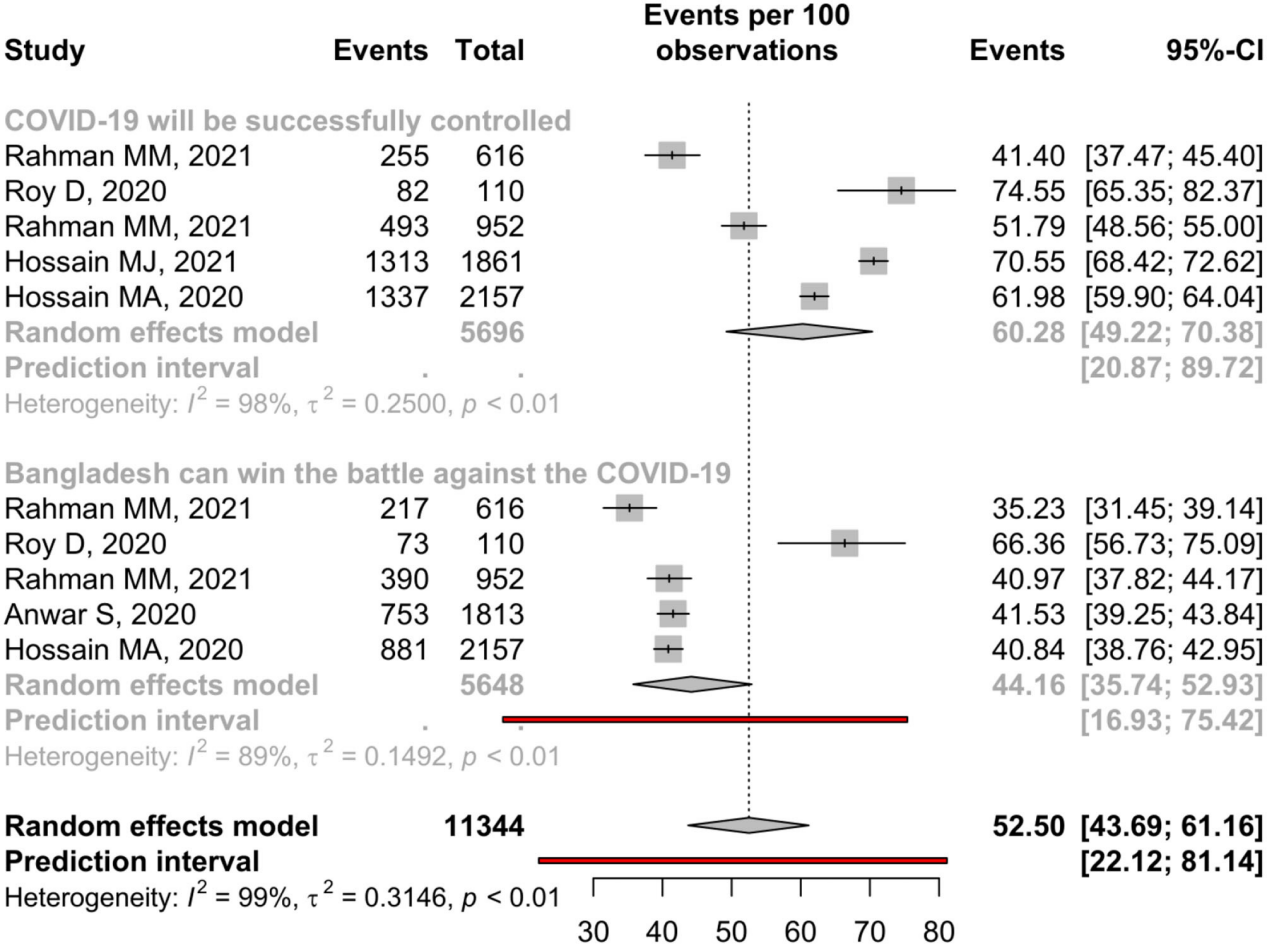
Attitude toward COVID-19 among Bangladeshi residents.

Five studies reported attitudes toward the control of COVID-19, and 60.28% (95%, CI: 49.22–70.38%) hold a positive attitude toward it (Table 3). In addition, three community-based and two institutional-based studies assessed this attitude among people with an estimated positive attitude of 58.47 and 63.06%, respectively (Supplementary Material). The forest is presented in Figure 5.

**Table 3:** Pooled prevalence of attitude toward COVID-19 among Bangladeshi residents.

**Attitudes toward COVID-19**	**No of study**	**Sample size**	**Percentage, (%)**	**95% CI**	**I^**2**^ (%)**	***p*** **-value**	**Eggers test**
COVID-19 will be successfully controlled	5	5,696	60.28	49.22–70.38	98.1	<0.01	0.72
Bangladesh can win the battle against the COVID-19	5	5,648	44.16	35.74–52.93	88.7	<0.01	0.37
Male	1	1,166	41.77	38.97–44.62			
Female	2	2,804	40.91	39.10–42.74	0.00	0.36	

Only 44.16% of people believe Bangladesh can win the battle against COVID-19, and males (41.77%) and females (40.91%) hold almost similar levels of positive attitude toward this (Table 3). In contrast, participants of institutional-based studies (53.12%) had a better attitude than the participants of community-based studies (39.71%) (Supplementary Material).

### Practice of COVID-19

**Figure 6:**
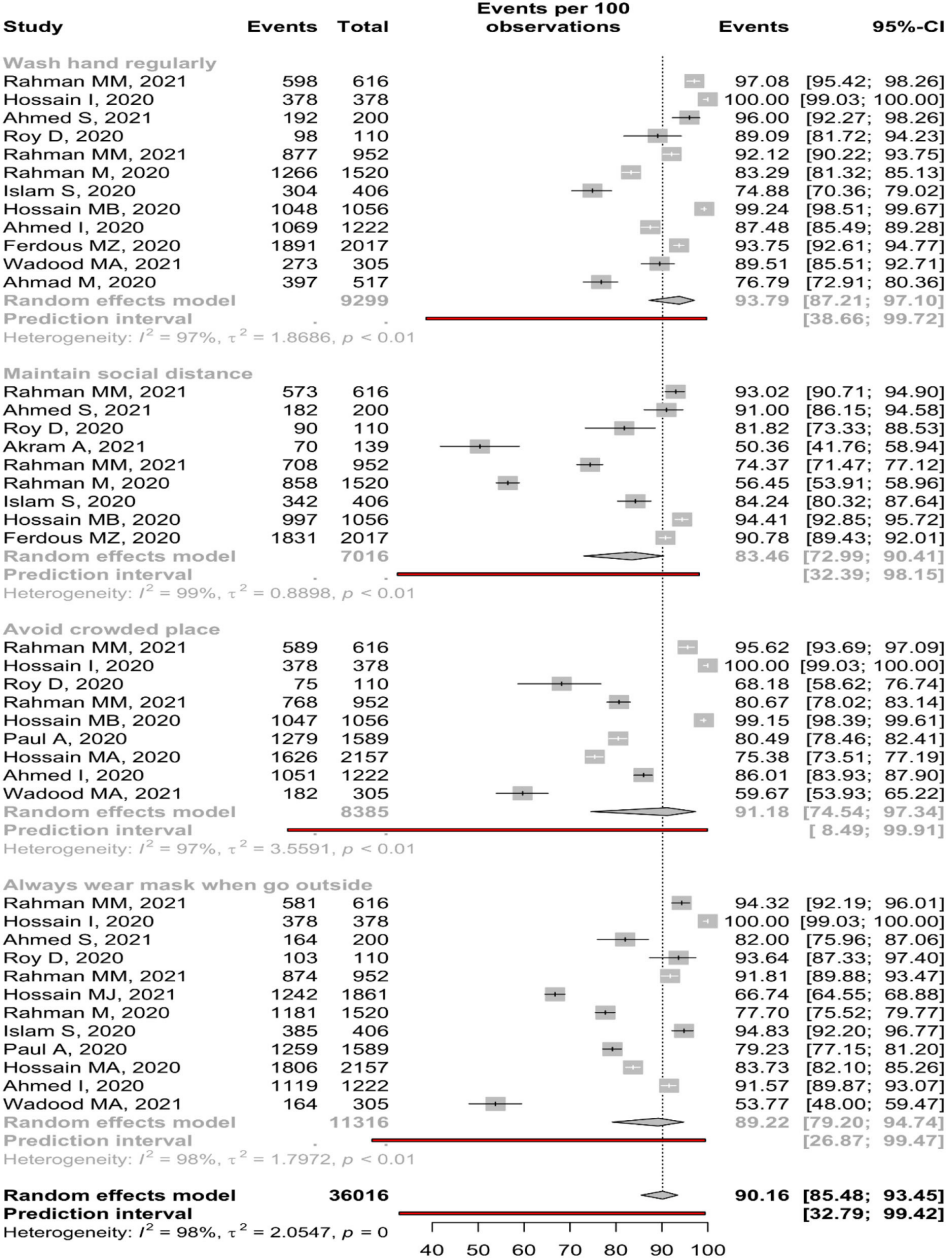
Practice about COVID-19 among Bangladeshi residents.

Only 12 studies reported handwashing practice; random-effect meta-analysis estimated 93.79% (95%, CI: 87.21–97.10%) participants regularly do this practice. A substantial amount of study heterogeneity was identified (I^2^ = 96.8%), whereas no small-study effect was found based on the Eggers tests (*p* = 0.09) (Table 4). The forest is presented in Figure 6. Male (91.30%, CI: 78.96–96.71%) and participants of IBCS (96.17%, CI: 85.54–99.07%) used to do this practice more frequently than female (90.53%, CI: 75.53–96.74%) and participants of CBCS (91.71%, CI: 81.28–96.57%) (Supplementary Material).

**Table 4:** Pooled prevalence of practice about COVID-19 among Bangladeshi residents.

**Practice of COVID-19**		**No of study**	**Sample size**	**Percentage, (%)**	**95% CI**	**I^**2**^ (%)**	***P*** **-value**	**Eggers test**
Wash hand regularly		12	9,299	93.79	87.21–97.10	96.8	<0.01	0.09
	Male	3	1,554	91.30	78.96–96.71	96.0	<0.01	
	Female	3	1,069	90.53	75.53–96>74	98.0	<0.01	
Maintain social distance		9	7,016	83.46	72.99–90.41	99.0	<0.01	0.17
	Male	3	1,554	87.13	85.37–88.71	27.7	0.25	
	Female	3	1,069	91.93	83.60–96.22	94.3	<0.01	
Avoid crowded place		9	8385	91.18	74.54–97.34	97.4	<0.01	0.17
	Male	1	1,166	70.15	67.46–72.71			
	Female	1	991	81.53	78.99–83.83			
Always wear mask when go outside		12	11,316	89.22	79.20–94.74	98.2	<0.01	0.08
	Male	3	1,514	90.54	80.39–95.71	88.0	<0.01	
	Female	3	1,249	85.19	75.85–91.33	87.2	<0.01	

Nine studies reported about maintaining social distance, and only 83.46 % (95%, CI: 72.99–90.41%) participants found to do this practice (Table 4). The forest is presented in Figure 6. Female (91.93%, CI: 83.60–96.22%) and participants of CBCS (87.29%, CI: 75.28–93.94%) used to do this practice more frequently than male (87.13% CI: 85.37–88.71%) and participants of IBCS (77.17%, CI: 59.92–88.43%) (Supplementary Material).

Random-effect meta-analysis revealed 91.18 % (95% CI: 74.54–97.34%, *p* < 0.01) participants avoid crowded place. A substantial amount of study heterogeneity was identified (I^2^ = 97.4%). In the Eggers tests, no small-study effect was found (*p* = 0.17) (Table 4). The forest is presented in Figure 6. About 91.86 % and 92.10% of participants of CBCS and IBCS avoid crowded places, respectively (Supplementary Material).

About 89.22% (95% CI: 79.20–94.74%) participants from 12 reported studies always wear mask during going outside (Table 4). The forest is presented in Figure 6. Male (90.54%, CI: 80.39–95.71%) and participants of IBCS (93.78%, CI: 65.43–99.18%) used to do this practice more frequently than female (85.19%, CI: 75.85–91.33%) and participants of CBCS (86.40%, CI: 78.03–91.91%) (Supplementary Material).

## Discussion

This systematic review and meta-analysis evaluated participants' overall knowledge of COVID-19 symptoms, transmission, treatment, and attitudes toward successfully controlling the COVID-19 pandemic. In addition, the COVID-19 control practices such as hand washing, wearing a mask, avoiding crowded places, and maintaining social distance were also evaluated in this study. The study included 18 articles, 12 of which used a community-based cross-sectional study design and six of which used an institutional-based cross-sectional study design.

Overall, a significant portion of the participants had knowledge about COVID-19 symptoms (89.87%, CI: 67.71–97.40%), whereas the symptoms like fever, dry cough, and respiratory signs were found to be known to the participants. But, less than half of the participants were not aware of diarrhea (39.24%) and weakness (49.28%) as the COVID-19 symptoms, and only 56.23% of people had positive knowledge about the headache; this may be due to those symptoms being less common among the COVID-19 infected individuals. In addition, a high percentage of participants were reported to know about COVID-19 transmission through respiratory droplets (92.09%) and the unavailability of its specific treatment (79.51%). In subgroup analysis, male gender had better knowledge than females, only in knowledge related to the symptom of dry cough, weakness, and diarrhea, while participants of the community-based studies were more informed about a sore throat, headache, diarrhea, dry cough, and fever as a symptom of COVID-19 than participants of the institutional-based studies. Those findings might denote that residents of Bangladesh have shallow and insufficient knowledge about some domains of the COVID-19 knowledge, where inappropriate and poor knowledge could potentially lead to a hover in the attempt to find medical support and, consequently, a hover in the early diagnosis and treatment (13, 14). Therefore, smattering knowledge about the disease impedes control and elimination due to negligence in disease prevention practices (33, 35). Therefore, it is suggested that the country's public health authority focuses on those knowledge aspects that are poorly reported while implementing health education programs.

Based on this review, it is found that only 44.16% of Bangladeshi residents thought that the country would win the battle against the COVID-19 pandemic. On the contrary, a higher level of positive attitude, that is, 72.39% of general people of Ethiopia, was enumerated in a meta-analysis of COVID-19 KAP studies (36), reflecting dissatisfaction about pandemic management in Bangladesh. Several challenges and probable poor management of the pandemic in the country can be the forces for driving such a poor attitude among the Bangladeshi residents (37). However, as the pandemic is new to the management authority of the country, such dissatisfaction can be resolved over time by growing up the competency of the respective authorities.

Regarding practice related to COVID-19, this study reveals that a significant portion of the participants maintained washing hands (93.79%), wearing a mask (89.22%), and avoiding crowded places (91.18%), but maintaining social distancing was not always possible for a large portion, especially for the participants of institution-based studies (77.17%). Furthermore, males more frequently wore masks than females, but females had other better prevention practices such as maintaining social distance and avoiding crowded places. This may be because males are more likely to visit outside the home, which leads them to wear a mask and wash hands more frequently than females. In addition, the higher mortality of COVID-19 rate among the Bangladeshi males (37, 38) could make them more concerned about following more precautious measures despite going outside (8).

Before concluding the importance and potential implications of this meta-analysis findings, several limitations are supposed to be noted. First of all, journal articles published in English were only taken into consideration, limiting other sources such as preprint articles. All of the included studies that were conducted in online data collection approaches, limiting the findings' generalizability to the entire country's population. Besides, some of the studies were excluded because of not meeting the inclusion criteria [e.g., ten-thousand nationwide data were collected in the study of Hosen et al. (16), which could help generate evidence based on such a wider-spaced population's findings]. Factors associated with the COVID-19 KAP were not considered in the present study. Despite those limitations, the potency of this meta-analysis lies in the compilation of the results of 18 papers, which accentuated the results of the individual studies and permitted to acquire a merged prevalence that generated a shred of stronger evidence about COVID-19 KAP among the general Bangladeshi population. The findings of this study are likely to aid Bangladeshi governments and policymakers in putting evidence into action by identifying gaps and emphasizing the importance of educating the less informed public about COVID-19 transmission.

## Conclusion

A number of KAP studies were undertaken in Bangladesh with populations from various categories, with notable variation in terms of gender, geography, occupation, and education. As a result, the study gives an overall KAP scenario for COVID-19 prophylaxis. This research should be taken into account by policymakers to underline the necessity of educating the less informed people about COVID-19 to restrict the spread of infection.

## Data Availability Statement

The original contributions presented in the study are included in the article/Supplementary Material, further inquiries can be directed to the corresponding author/s.

## Author Contributions

AR and MM conceptualized the study. AR and RR did data extraction, where AR and Fa-M performed formal analysis. AR and SJ wrote the first draft, which has been extensively reviewed and edited by MM. Later on, further review and subsequent edits were performed by AR, AH, Fa-M, and MM. AR, Fa-M, and MM contributed to the study's methodology. AR managed resources and software with the help of Fa-M. AR, AH, and MM supervised the project. All authors read and finally approved the submission for publication.

## Conflict of Interest

The authors declare that the research was conducted in the absence of any commercial or financial relationships that could be construed as a potential conflict of interest.

## Publisher's Note

All claims expressed in this article are solely those of the authors and do not necessarily represent those of their affiliated organizations, or those of the publisher, the editors and the reviewers. Any product that may be evaluated in this article, or claim that may be made by its manufacturer, is not guaranteed or endorsed by the publisher.
