# Supplementary material for: Knowledge, Attitudes, and Practices Toward the Prevention of COVID-19 in Bangladesh: A Systematic Review and Meta-Analysis
Source: Front Med (Lausanne). 2022 Jun 6;9:856156. doi: 10.3389/fmed.2022.856156 (PMC9208617; doi:10.3389/fmed.2022.856156)
Supplement: Supplementary file 1 [file Data_Sheet_1.docx]

**Supplementary Table 1: Algorithm for study search to identify published articles on the knowledge, attitude and practice of COVID-19 among Bangladeshi residents**

| Search function | Search term |
| --- | --- |
| Any of | "Knowledge" OR "Attitude" OR "Perception" OR "Practice" |
| AND Any of | "corona virus" OR "novel corona virus" OR "Covid- 19" OR "severe acute respiratory syndrome" |
| AND | "Bangladesh" |

**Supplementary Table 2: Data that were extracted from the selected articles**

| **Category** | **Data to be collected** |
| --- | --- |
| **General** | Name of the first author |
|  | Year of publication |
|  | No. of participants |
|  | Mean age of the participants |
|  | Gender (percentage of male/female participants) |
|  | Sampling date |
|  | Focus group |
|  | Questionnaire administration |
| **Knowledge** | |
| Knowledge about Covid-19 Symptoms | Symptoms of Covid |
|  | Fever |
|  | Dry cough |
|  | Respiratory sign |
|  | Weakness |
|  | Diarrhea |
|  | Headache |
| Knowledge about Covid-19 Transmission | Spread through respiratory droplet |
| Knowledge about Covid-19 Treatment | No-specific treatment available |
| **Attitude** | COVID-19 will be successfully controlled |
|  | Bangladesh can win the battle against the COVID-19 |
| **Practice** | Wash hand regularly |
|  | Maintain social distance |
|  | Avoid crowded place |
|  | Always wear mask when go outside |

**Supplement Table 3:Study quality assessment questions**

| **Risk of bias items** | | | |
| --- | --- | --- | --- |
| **External Validity** | | **Internal Validity** | |
| 1 | Was the study's target population a close representation of the national population in relation to relevant variables, e.g. age, sex, occupation? | 5 | Were data collected directly from the subjects? |
| 2 | Was the sampling frame a true or close representation of the target population? | 6 | Was the research purpose and study design reasonable? |
| 3 | Was some form of random selection used to select the sample, OR, was a census undertaken? | 7 | Was the study instrument that measured the parameter of interest shown to have reliability and validity? |
| 4 | Was the likelihood of Non-response rate reported? | 8 | Was the same mode of data collection used for all subjects? |
|  |  | 9 | Was the length of the shortest prevalence period for the parameter of interest appropriate? |
|  |  | 10 | Were the numerator( s) and denominato r(s) for the parameter of interest appropriate? |

**Supplement Table 4:Study quality assessment scores**

| **Risk of bias items** | **External Validity** | | | | **Internal Validity** | | | | | | **Overall risk of study bias** |
| --- | --- | --- | --- | --- | --- | --- | --- | --- | --- | --- | --- |
|  | **1** | **2** | **3** | **4** | **5** | **6** | **7** | **8** | **9** | **10** |  |
| Rahman MM, 2021 [21] | No | Yes | Yes | No | Yes | Yes | Yes | Yes | Yes | Yes | Low |
| Hossain I, 2020 [30] | No | Yes | Yes | No | Yes | Yes | No | Yes | Yes | Yes | Low |
| Pervez S, 2021 [22] | No | No | Yes | No | Yes | Yes | Yes | Yes | Yes | Yes | Low |
| Ahmed S, 2021 [31] | No | No | Yes | No | Yes | Yes | Yes | Yes | Yes | Yes | Low |
| Roy D, 2020 [32] | No | No | No | No | Yes | Yes | Yes | Yes | Yes | Yes | Moderate |
| Akram A, 2021 [33] | No | No | No | Yes | Yes | Yes | Yes | Yes | Yes | Yes | Low |
| Rahman MM, 2021 [2] | No | Yes | Yes | No | Yes | Yes | Yes | Yes | Yes | Yes | Low |
| Hossain MJ, 2021 [23] | No | Yes | Yes | No | Yes | Yes | Yes | No | Yes | Yes | Low |
| Rahman M, 2020 [24] | No | Yes | Yes | No | Yes | Yes | Yes | Yes | Yes | Yes | Low |
| Islam S, 2020 [25] | No | Yes | No | No | Yes | Yes | Yes | Yes | Yes | Yes | Low |
| Anwar S, 2020 [9] | No | Yes | No | Yes | Yes | Yes | Yes | No | Yes | Yes | Low |
| Hossain MB, 2020 [26] | No | Yes | Yes | Yes | Yes | Yes | Yes | Yes | Yes | Yes | Low |
| Paul A, 2020 [27] | No | Yes | Yes | No | Yes | Yes | Yes | Yes | Yes | Yes | Low |
| Hossain MA, 2020 [8] | No | Yes | Yes | No | Yes | Yes | Yes | Yes | Yes | Yes | Low |
| Ahmed I, 2020 [28] | No | Yes | No | No | Yes | Yes | No | Yes | Yes | Yes | Moderate |
| Ferdous MZ, 2020 [6] | No | Yes | Yes | Yes | Yes | Yes | No | Yes | Yes | Yes | Low |
| Wadood MA, 2021 [1] | No | No | No | No | Yes | Yes | Yes | Yes | Yes | Yes | Moderate |
| Ahmad M, 2020 [29] | No | Yes | Yes | No | Yes | Yes | No | Yes | Yes | Yes | Low |

**Supplementary Table 5: Subgroup analysis of Covid-19 knowledge**

| Knowledge | | No of study | Sample size | Percentage (%) | 95% CI | I^2^  (%) | P-value |
| --- | --- | --- | --- | --- | --- | --- | --- |
| **Knowledge about Covid-19 Symptoms** | | | | | | | |
| **Symptoms of Covid** | | | | | | | |
| Study Design | CBCS | 5 | 6438 | 68.74 | 50.40-82.64 | 99.3 | < 0.01 |
|  | IBCS | 4 | 1579 | 98.40 | 85.25-99.85 | 95.4 | < 0.01 |
| **Fever** | | | | | | | |
| Gender | Male | 3 | 521 | 89.64 | 86.71-91.98 | 15.7 | 0.31 |
|  | Female | 3 | 400 | 91.93 | 86.52-95.29 | 65.8 | 0.05 |
| Study Design | CBCS | 4 | 3297 | 94.28 | 90.42-96.64 | 93.5 | < 0.01 |
|  | IBCS | 2 | 505 | 91.49 | 88.71-93.62 | 0 | 0.75 |
| **Dry cough** | | | | | | | |
| Gender | Male | 3 | 521 | 82.05 | 77.37-85.94 | 61.3 | 0.08 |
|  | Female | 3 | 400 | 77.14 | 68.48-83.99 | 79.6 | < 0.01 |
| Study Design | CBCS | 4 | 3297 | 88.12 | 78.95-93.61 | 97.0 | < 0.01 |
|  | IBCS | 2 | 505 | 79.21 | 75.45-82.53 | 51.5 | 0.15 |
| **Respiratory sign** | | | | | | | |
| Gender | Male | 3 | 521 | 79.36 | 55.29-92.28 | 97.3 | < 0.01 |
|  | Female | 3 | 400 | 88.61 | 42.69-98.78 | 97.8 | < 0.01 |
| Study Design | CBCS | 4 | 3297 | 84.71 | 59.12-95.50 | 99.3 | < 0.01 |
|  | IBCS | 2 | 505 | 88.01 | 72.36-95.37 | 95.0 | < 0.01 |
| **Weakness** | | | | | | | |
| Gender | Male | 3 | 521 | 46.12 | 20.47-74.00 | 98.1 | < 0.01 |
|  | Female | 3 | 400 | 33.14 | 7.24-75.89 | 97.7 | < 0.01 |
| Study Design | CBCS | 4 | 3297 | 46.42 | 21.81-72.91 | 99.0 | < 0.01 |
|  | IBCS | 1 | 200 | 60.50 | 53.56-67.04 |  |  |
| **Diarrhea** | | | | | | | |
| Gender | Male | 3 | 521 | 37.24 | 13.60-69.12 | 97.9 | < 0.01 |
|  | Female | 3 | 400 | 26.26 | 4.98-70.77 | 97.0 | < 0.01 |
| Study Design | CBCS | 4 | 3297 | 42.43 | 16.09- 73.90 | 99.2 | < 0.01 |
|  | IBCS | 2 | 505 | 33.06 | 11.99-64.16 | 98.7 | < 0.01 |
| **Headache** | | | | | | | |
| Study Design | CBCS | 2 | 1371 | 56.57 | 47.36- 65.36 | 94.1 | < 0.01 |
|  | IBCS | 1 | 305 | 55.08 | 49.46-60.58 |  |  |
| **Sore throat** | | | | | | | |
| Study Design | CBCS | 3 | 2982 | 78.88 | 44.61-94.54 | 99.6 | < 0.01 |
|  | IBCS | 1 | 305 | 49.84 | 44.25-55.43 |  |  |
| **Knowledge about Covid-19 Transmission** | | | | | | | |
| **Spread through respiratory droplet** | | | | | | | |
| Gender | Male | 3 | 1554 | 85.67 | 65.11-95.04 | 95.6 | < 0.01 |
|  | Female | 3 | 1069 | 85.69 | 61.45-95.74 | 94.7 | < 0.01 |
| Study Design | CBCS | 5 | 1945 | 85.82 | 66.41-94.88 | 86.6 | < 0.01 |
|  |  | 5 | 6588 | 95.82 | 92.50- 97.70 | 99.4 | < 0.01 |
| **Knowledge about Covid-19 Treatment** | | | | | | | |
| **No-specific treatment available** | | | | | | | |
| Gender | Male | 1 | 1206 | 80.18 | 77.84-82.34 |  |  |
|  | Female | 2 | 2624 | 82.36 | 80.85-83.77 | 0.00 | 0.38 |
| Study Design | CBCS | 8 | 11007 | 74.01 | 65.83-80.80 | 98.2 | < 0.01 |
|  | IBCS | 3 | 627 | 95.84 | 11.58-99.98 | 97.4 | < 0.01 |

**Supplementary Table 6: Subgroup analysis of Covid-19 attitude**

| Attitude | | No of study | Sample size | Percentage (%) | 95% CI | I^2^  ^(%)^ | P-value |
| --- | --- | --- | --- | --- | --- | --- | --- |
| **COVID-19 will be successfully controlled** | | | | | | | |
| Study Design | CBCS | 3 | 4634 | 58.47 | 44.22-71.44 | 98.8 | < 0.01 |
|  | IBCS | 2 | 1062 | 63.06 | 45.87-77.48 | 94.8 | < 0.01 |
| **Bangladesh can win the battle against the COVID-19** | | | | | | | |
| Gender | Male | 1 | 1166 | 41.77 | 38.97-44.62 |  |  |
|  | Female | 2 | 2804 | 40.91 | 39.10-42.74 | 0.00 | 0.36 |
| Study Design | CBCS | 3 | 4586 | 39.71 | 36.92-42.57 | 74.9 | 0.02 |
|  | IBCS | 2 | 1062 | 53.12 | 35.37-70.12 | 95.9 | < 0.01 |

**Supplementary Table 7: Subgroup analysis of Covid-19 practice**

| Practice | | No of study | Sample size | Percentage (%) | 95% CI | I^2^  ^(%)^ | P-value |
| --- | --- | --- | --- | --- | --- | --- | --- |
| **Wash hand regularly** | | | | | | | |
| Gender | Male | 3 | 1554 | 91.30 | 78.96-96.71 | 96.0 | < 0.01 |
|  | Female | 3 | 1069 | 90.53 | 75.53- 96.74 | 98.0 | < 0.01 |
| Study Design | CBCS | 7 | 7354 | 91.71 | 81.28- 96.57 | 98.0 | < 0.01 |
|  | IBCS | 5 | 1945 | 96.17 | 85.54-99.07 | 48.3 | < 0.01 |
| **Maintain social distance** | | | | | | | |
| Gender | Male | 3 | 1554 | 87.13 | 85.37- 88.71 | 27.7 | 0.25 |
|  | Female | 3 | 1069 | 91.93 | 83.60-96.22 | 94.3 | < 0.01 |
| Study Design | CBCS | 5 | 5615 | 87.29 | 75.28 - 93.94 | 99.5 | < 0.01 |
|  | IBCS | 4 | 1401 | 77.17 | 59.92 -88.43 | 95.5 | < 0.01 |
| **Avoid crowded place** | | | | | | | |
| Gender | Male | 1 | 1166 | 70.15 | 67.46- 72.71 |  |  |
|  | Female | 1 | 991 | 81.53 | 78.99-83.83 |  |  |
| Study Design | CBCS | 5 | 6604 | 91.86 | 77.86-97.31 | 98.3 | < 0.01 |
|  | IBCS | 4 | 1745 | 92.10 | 37.38-99.56 | 94.6 | < 0.01 |
| **Always wear mask when go outside** | | | | | | | |
| Gender | Male | 3 | 1514 | 90.54 | 80.39-95.71 | 88.0 | < 0.01 |
|  | Female | 3 | 1249 | 85.19 | 75.85 - 91.33 | 87.2 | < 0.01 |
| Study Design | CBCS | 7 | 9371 | 86.40 | 78.03- 91.91 | 98.6 | < 0.01 |
|  | IBCS | 5 | 1945 | 93.78 | 65.43- 99.18 | 98.0 | < 0.01 |

**
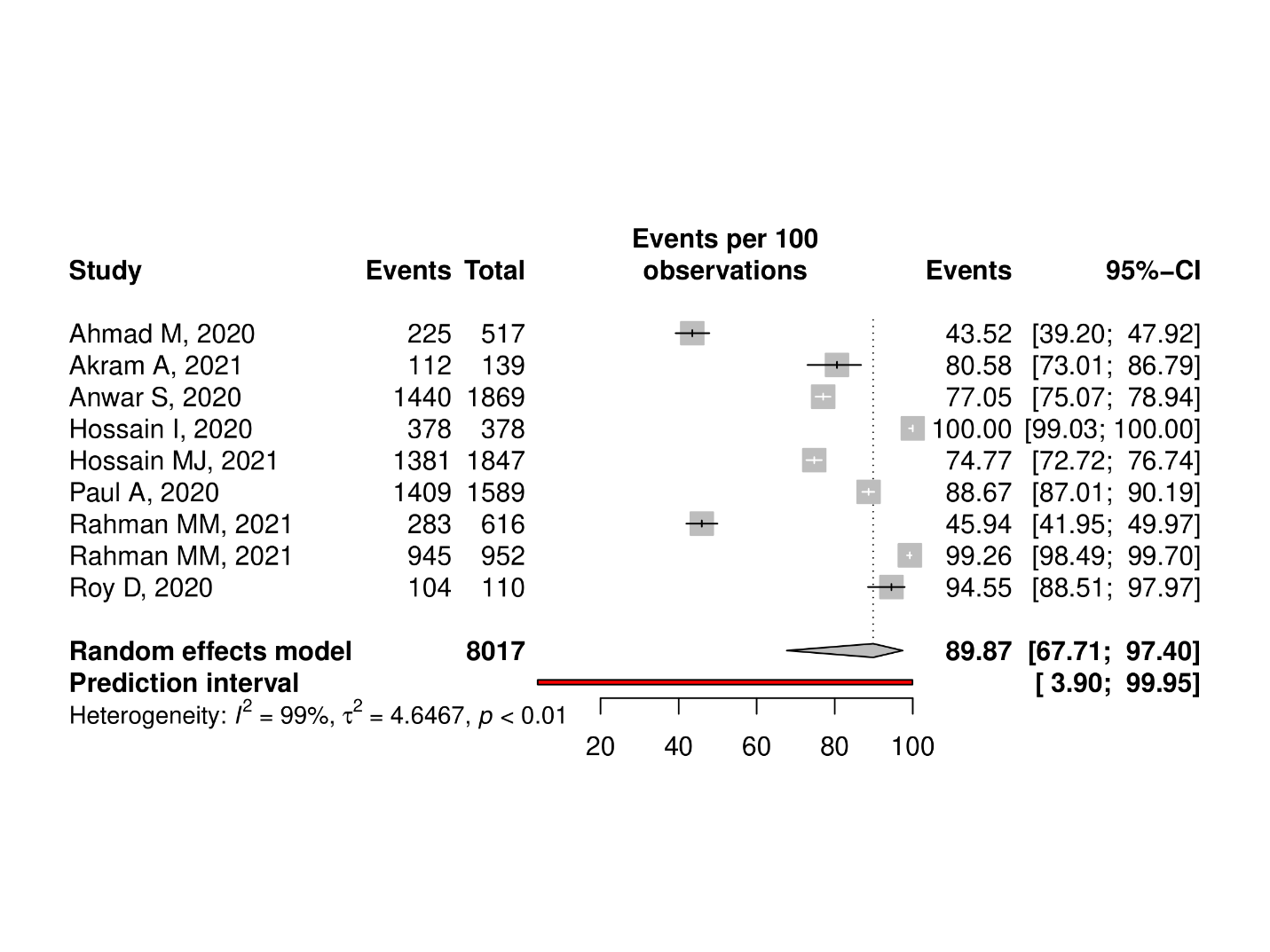
**

**Supplementary Figure 1: Knowledge of Participants on overall COVID-19 symptoms**

**
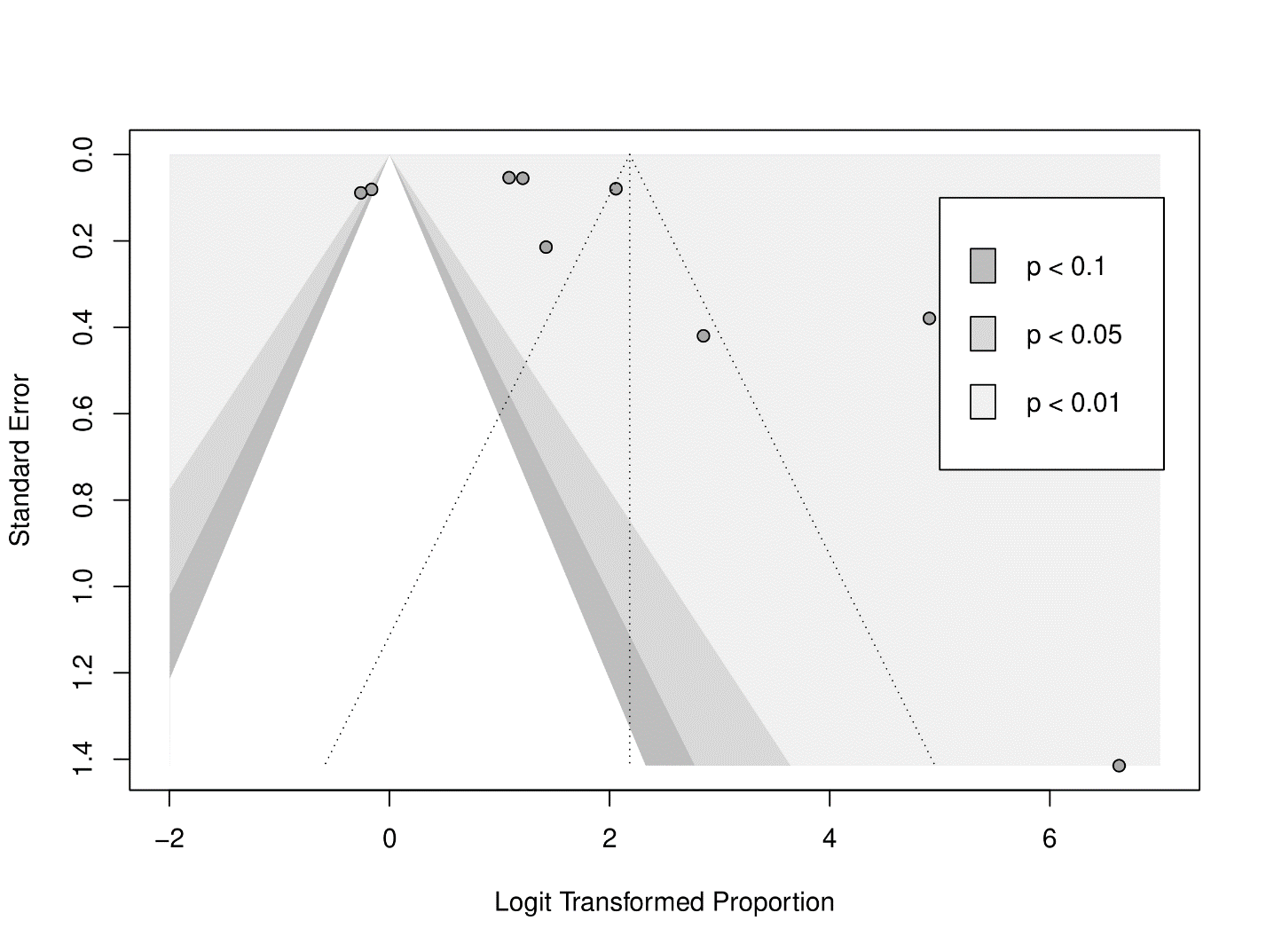
**

**Supplementary Figure 2: Funnel plot of Knowledge of Participants on overall COVID-19 symptoms**

**
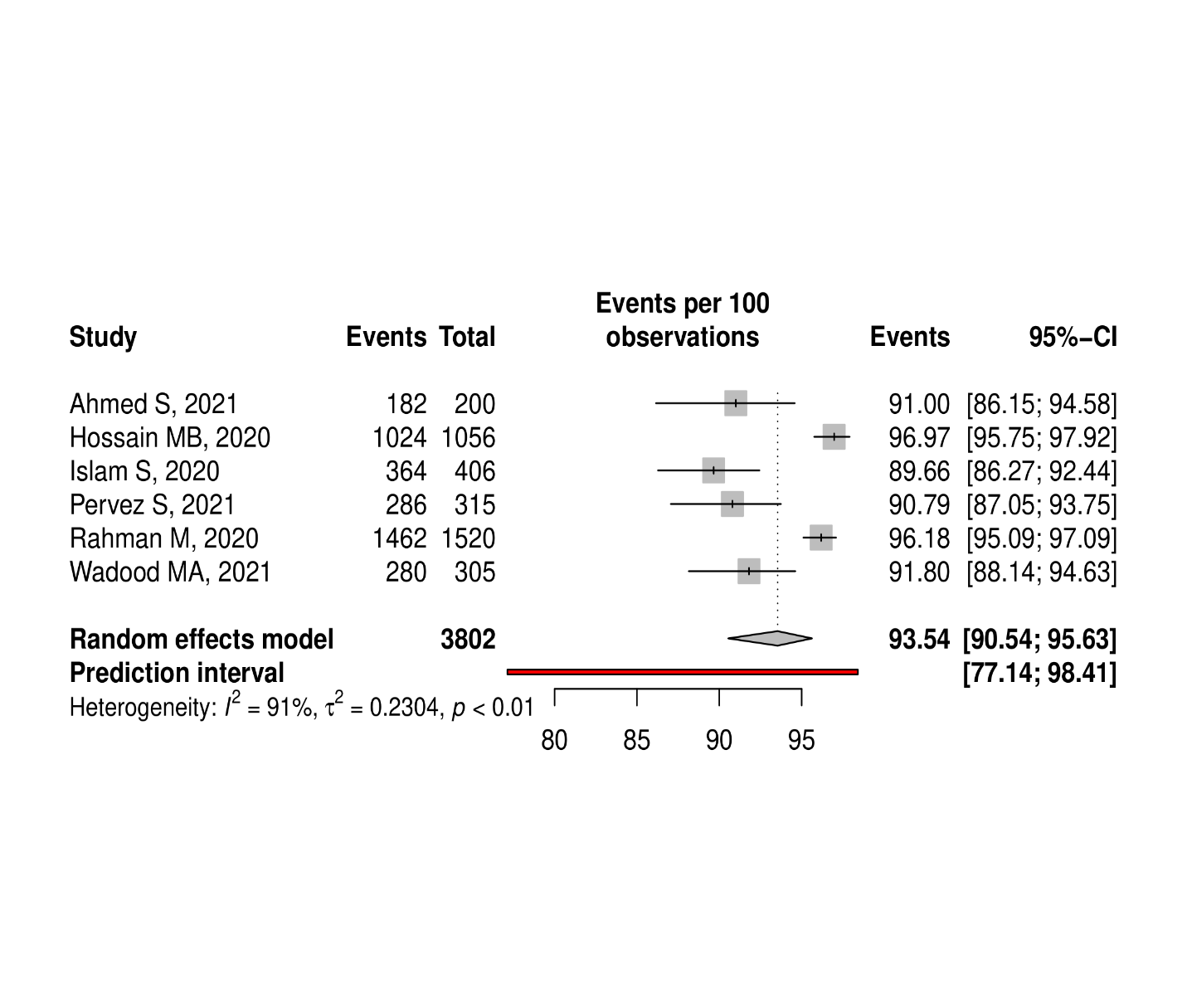
**

**Supplementary Figure 3: Knowledge of participants about Fever as a symptom of Covid-19**

**
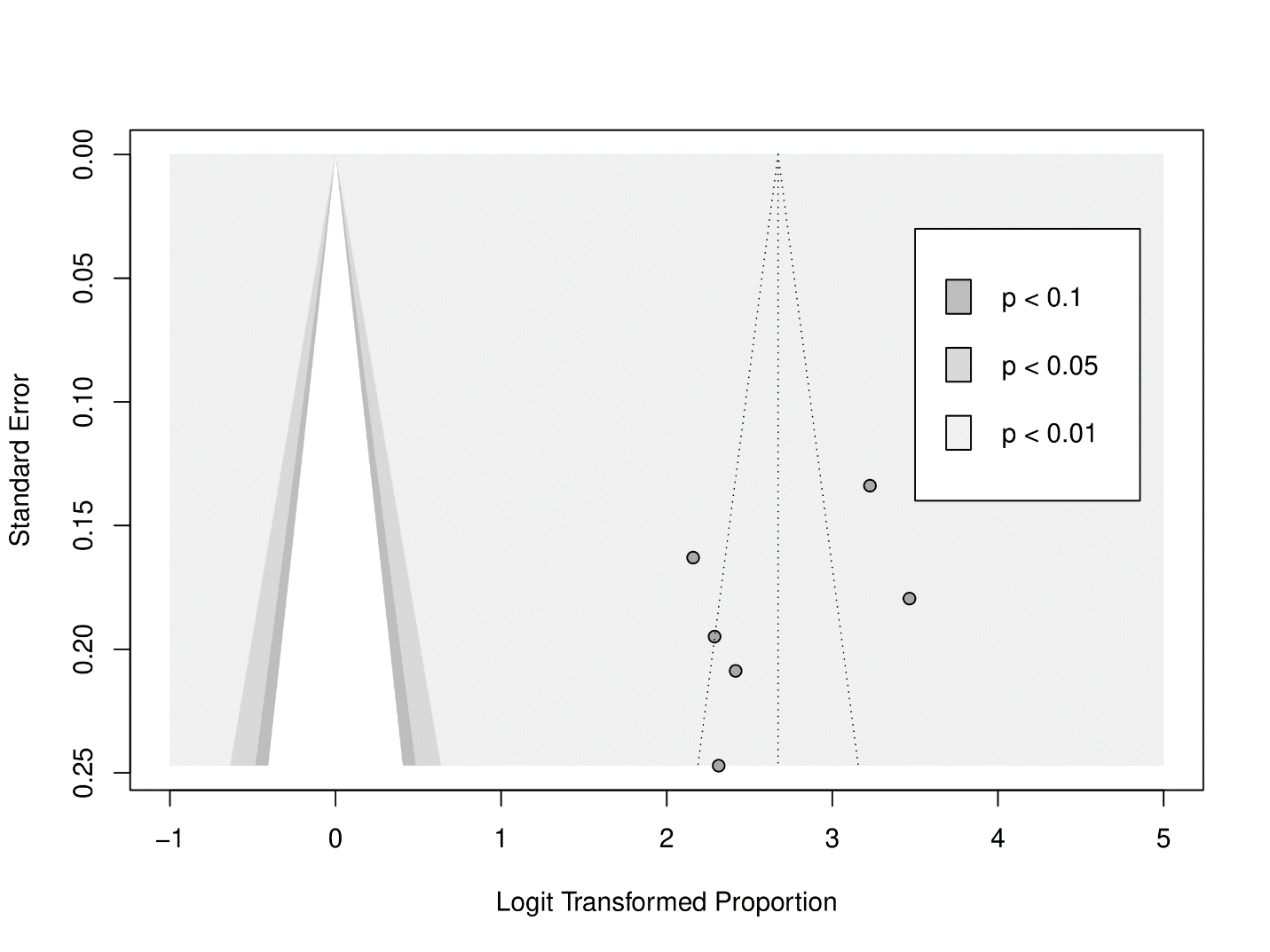
**

**Supplementary Figure 4: Funnel plot of Knowledge of participants about Fever as a symptom of Covid-19**

**
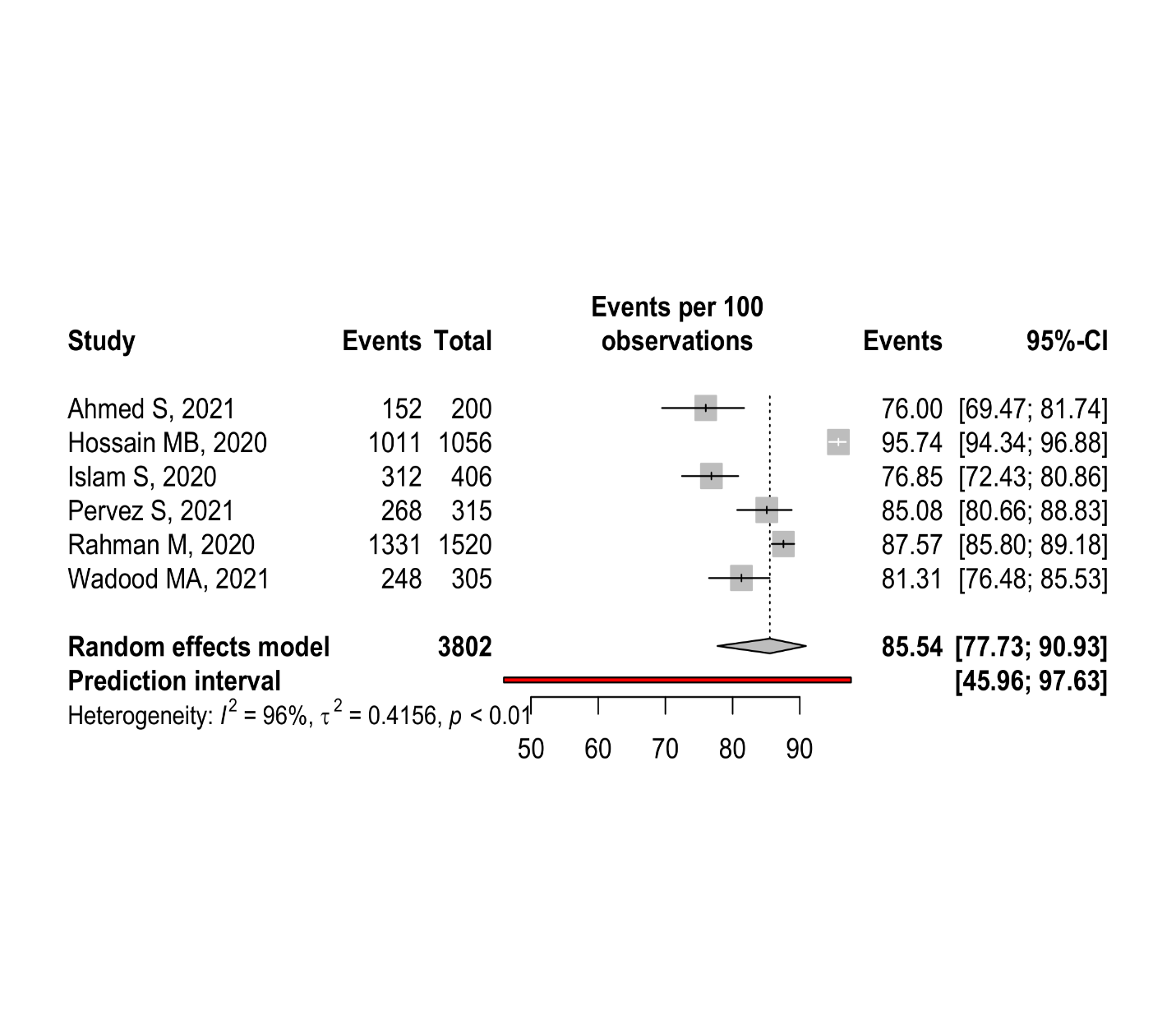
**

**Supplementary Figure 5: Knowledge of participants about Dry cough as a symptom of Covid-19**

**
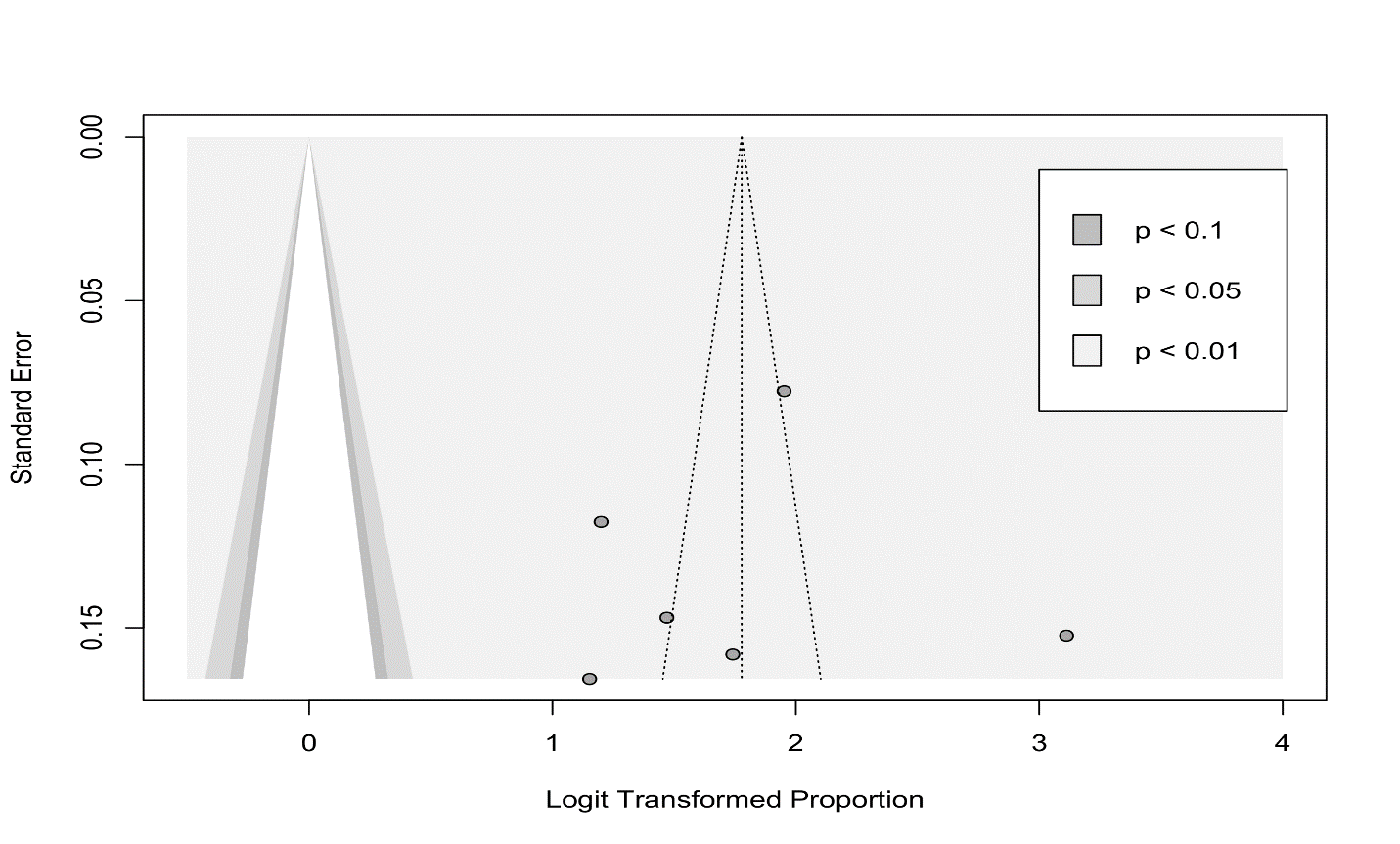
**

**Supplementary Figure 6: Funnel plot of Knowledge of participants about Dry cough as a symptom of Covid-19**

**
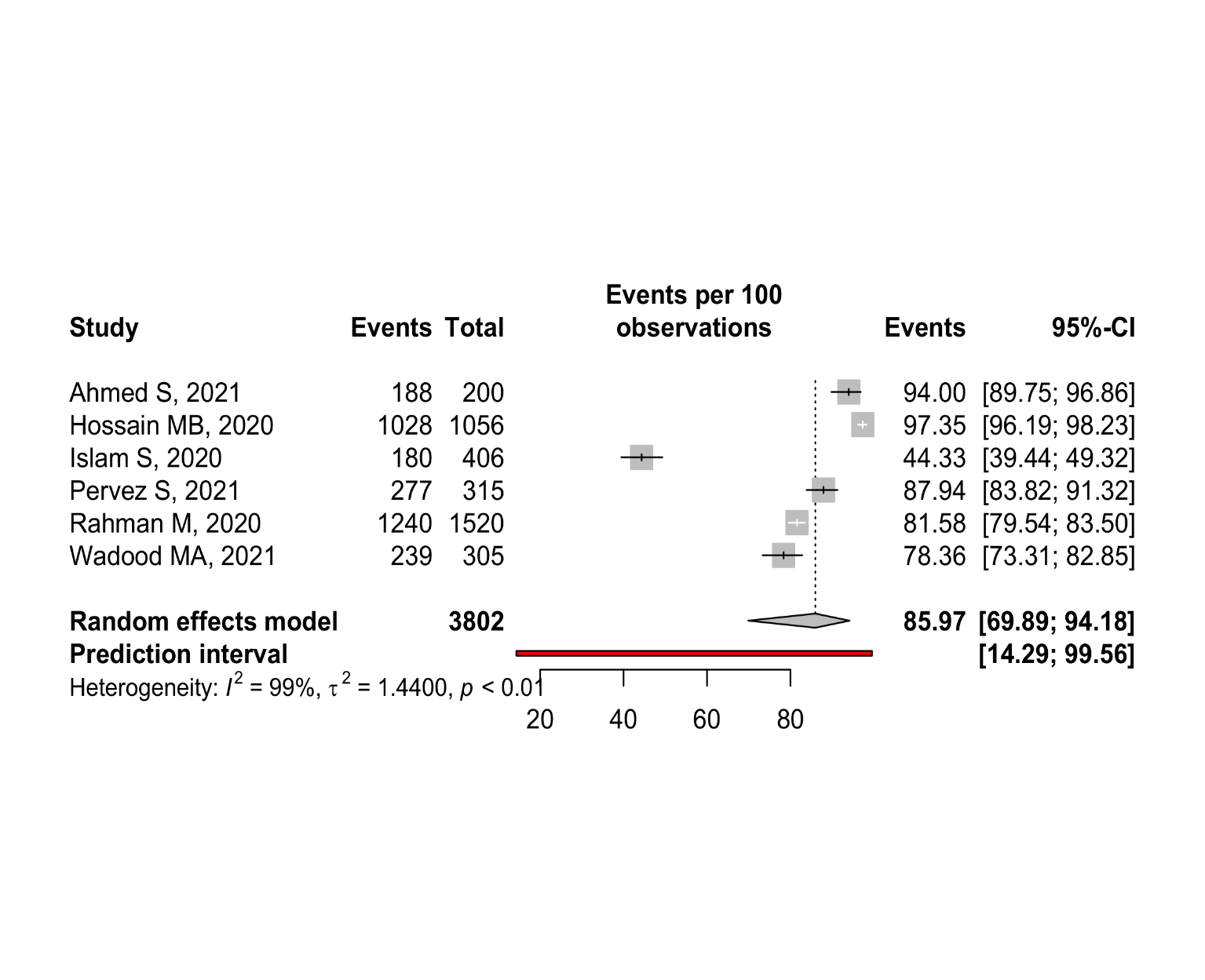
**

**Supplementary Figure 7: Knowledge of participants about Respiratory signs as a symptom of Covid-19**

**
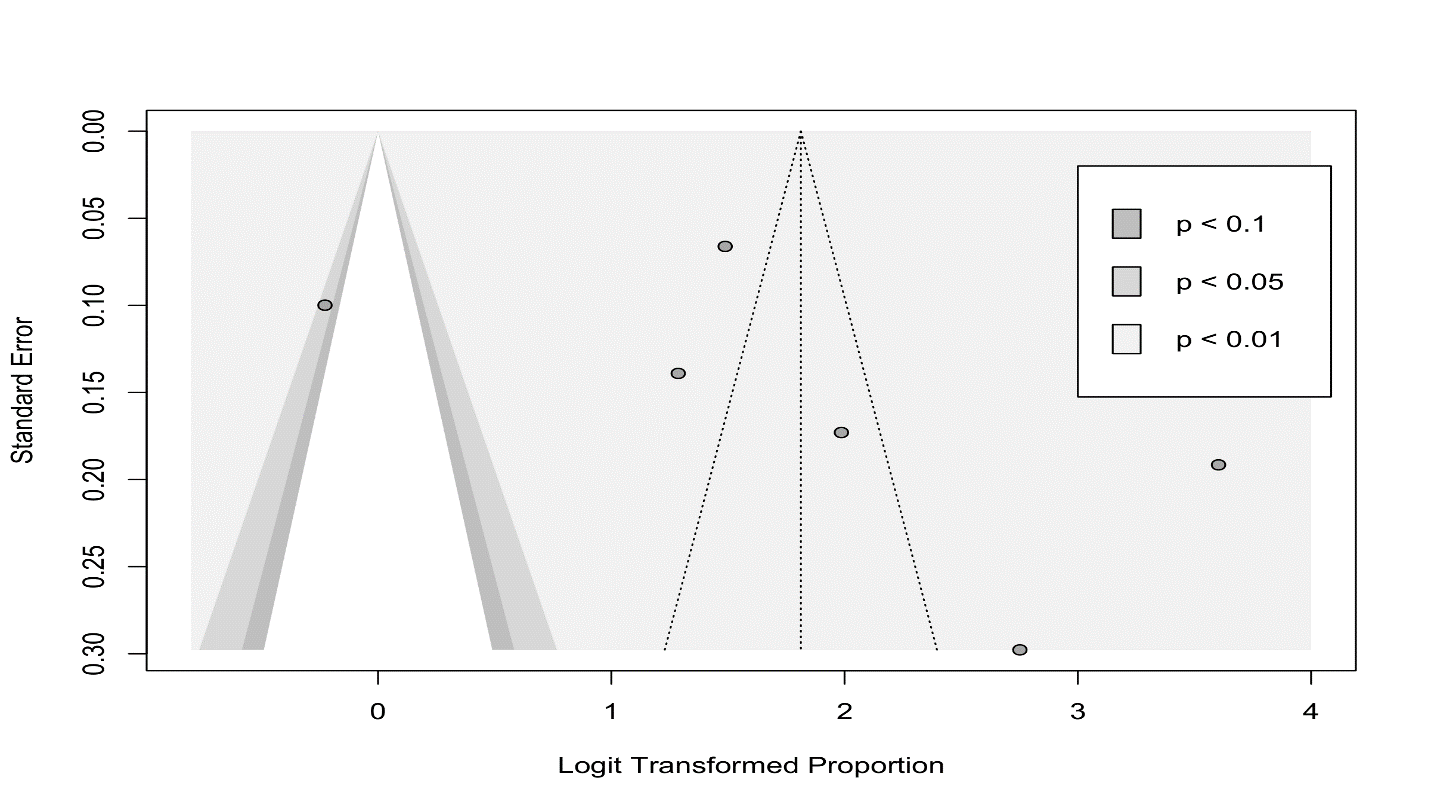
**

**Supplementary Figure 8: Funnel plot of Knowledge of participants about Respiratory signs as a symptom of Covid-19**

**
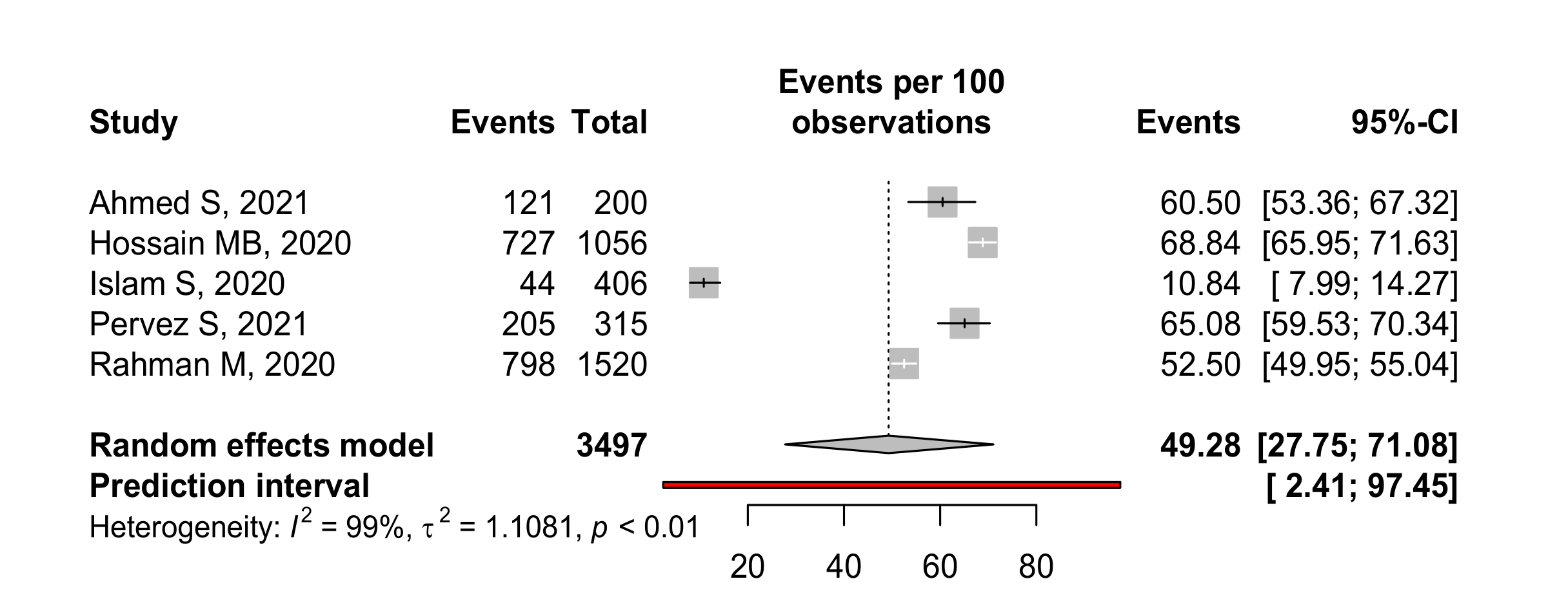
**

**Supplementary Figure 9: Knowledge of participants about weakness as a symptom of Covid-19**

**
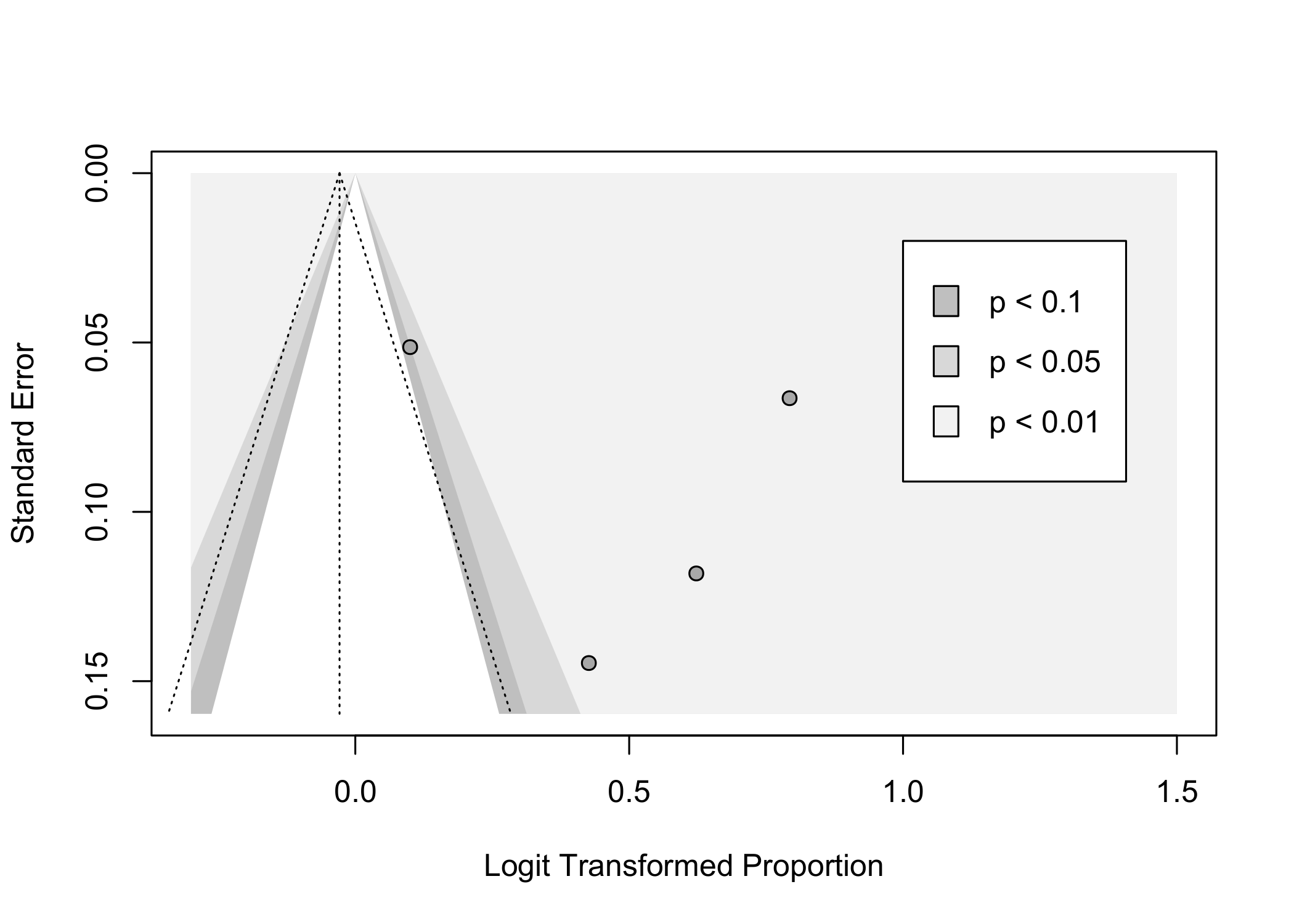
**

**Supplementary Figure 10: Funnel plot of Knowledge of participants about weakness as a symptom of Covid-19**

**
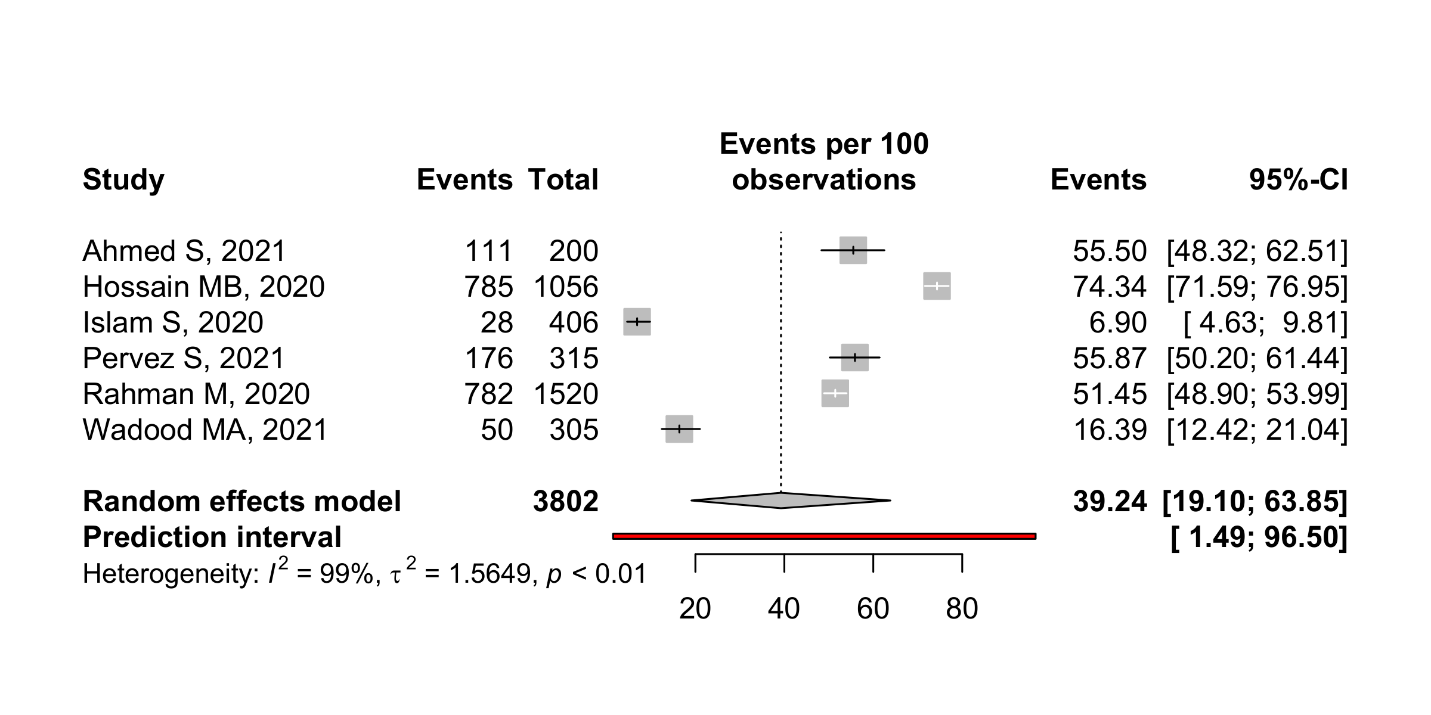
**

**Supplementary Figure 11: Knowledge of participants about Diarrhea as a symptom of Covid-19**

**
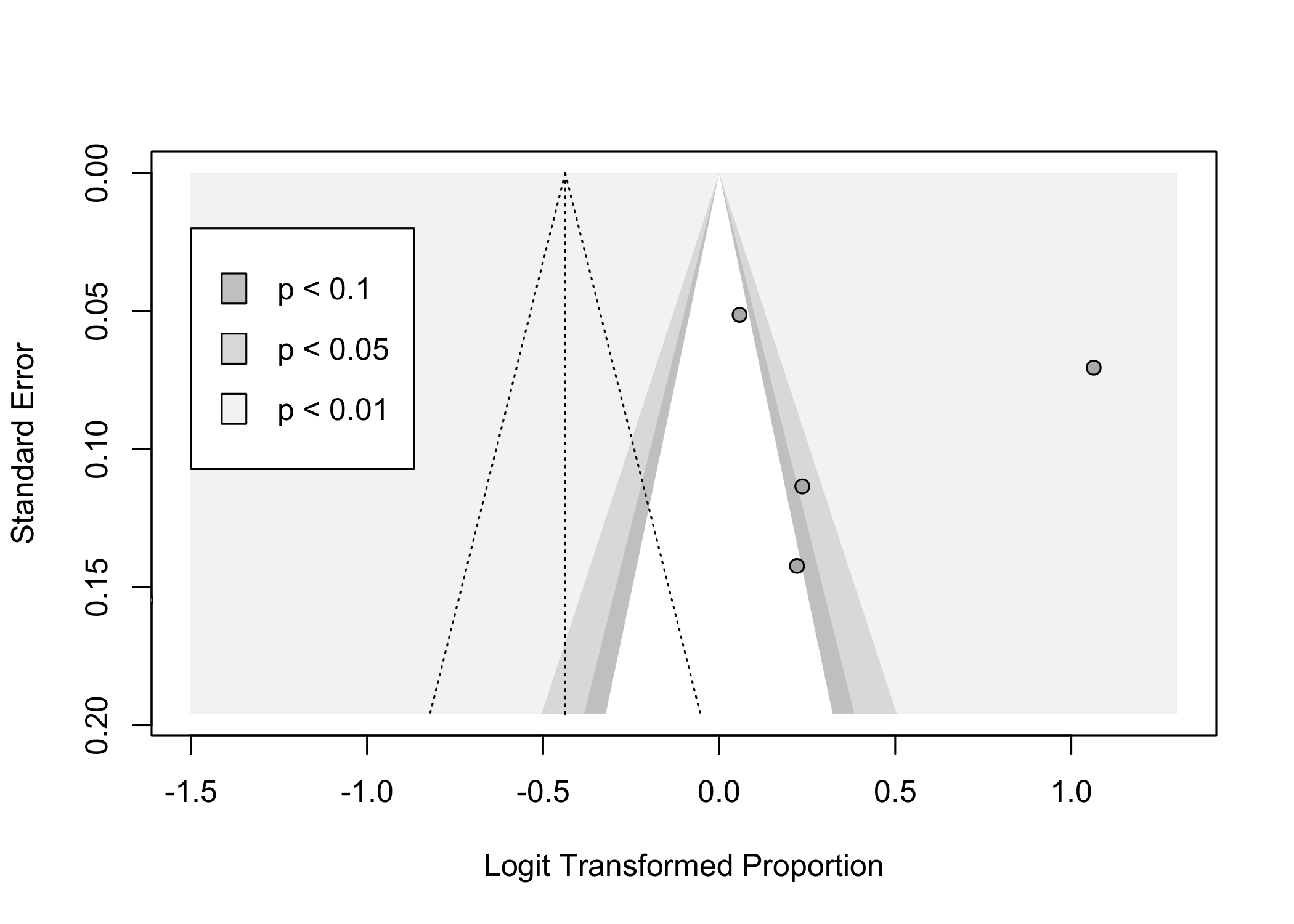
**

**Supplementary Figure 12: Funnel plot of Knowledge of participants about Diarrhea as a symptom of Covid-19**

**
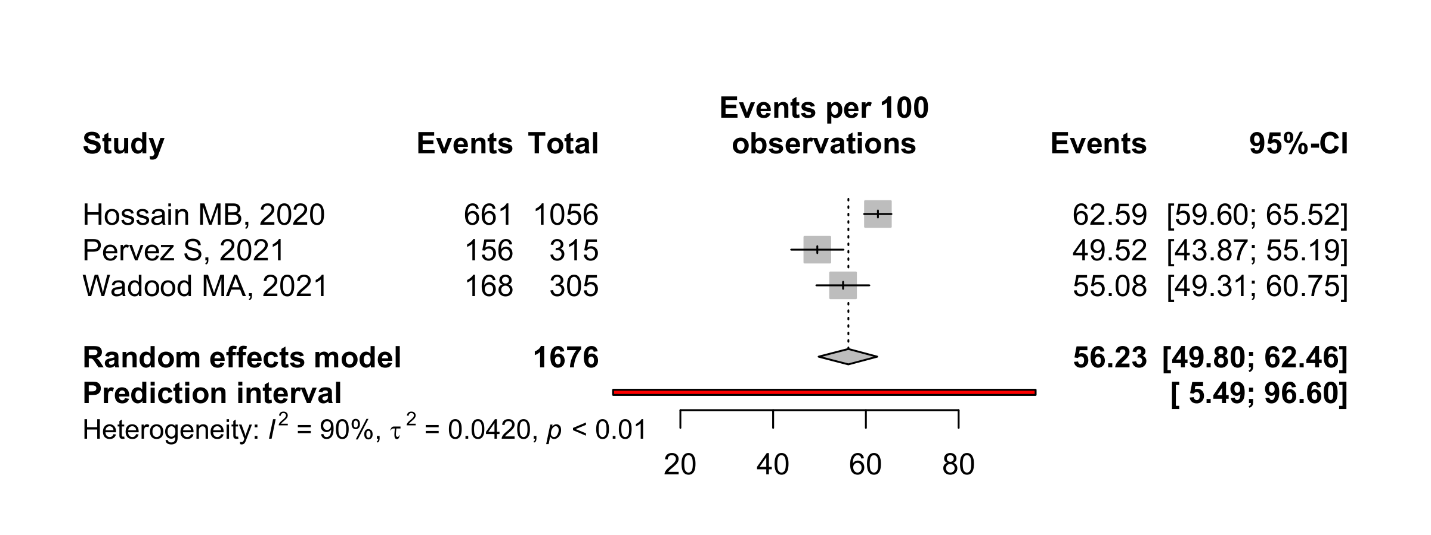
**

**Supplementary Figure 13: Knowledge of participants about Headache as a symptom of Covid-19**

**
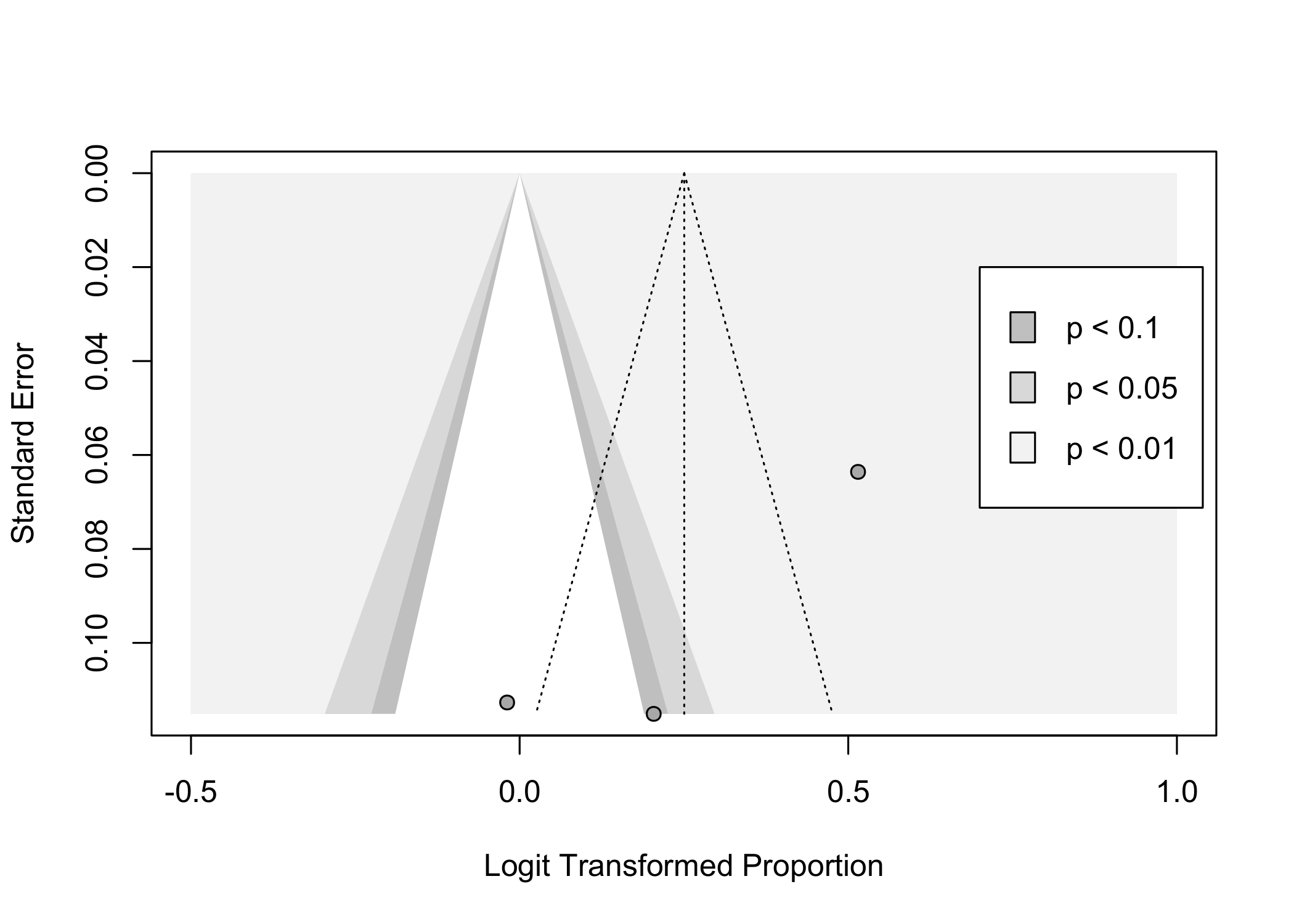
**

**Supplementary Figure 14: Funnel plot of Knowledge of participants about Headache as a symptom of Covid-19**

**
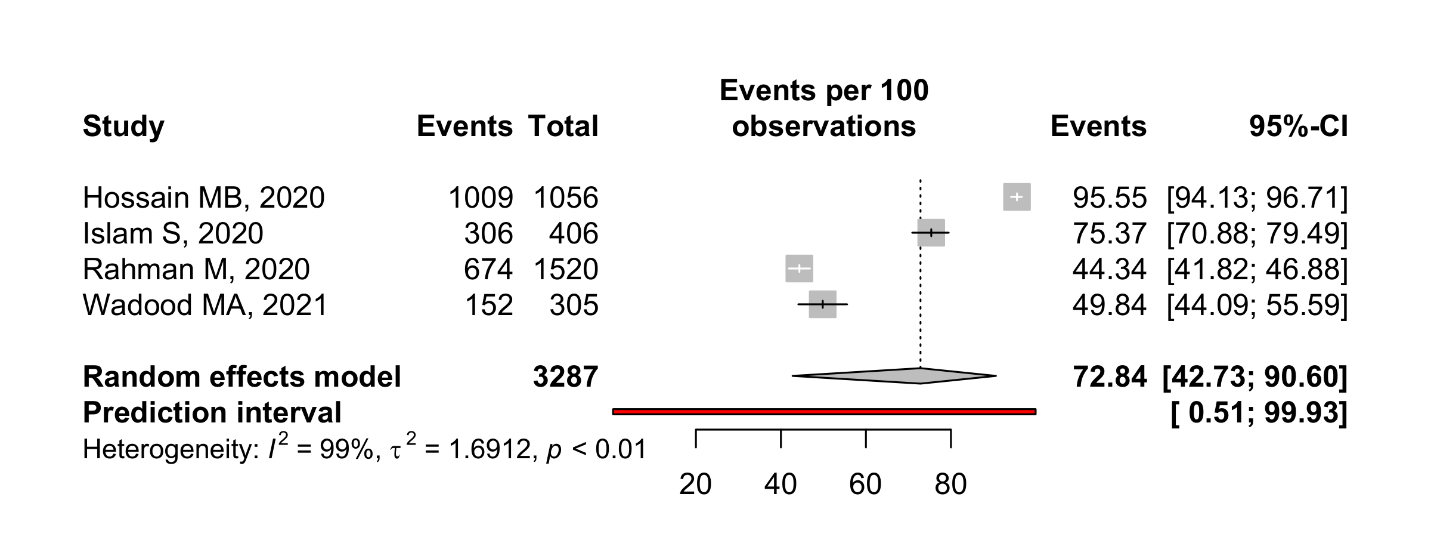
**

**Supplementary Figure 15: Knowledge of participants about Sore throat as a symptom of Covid-19**

**
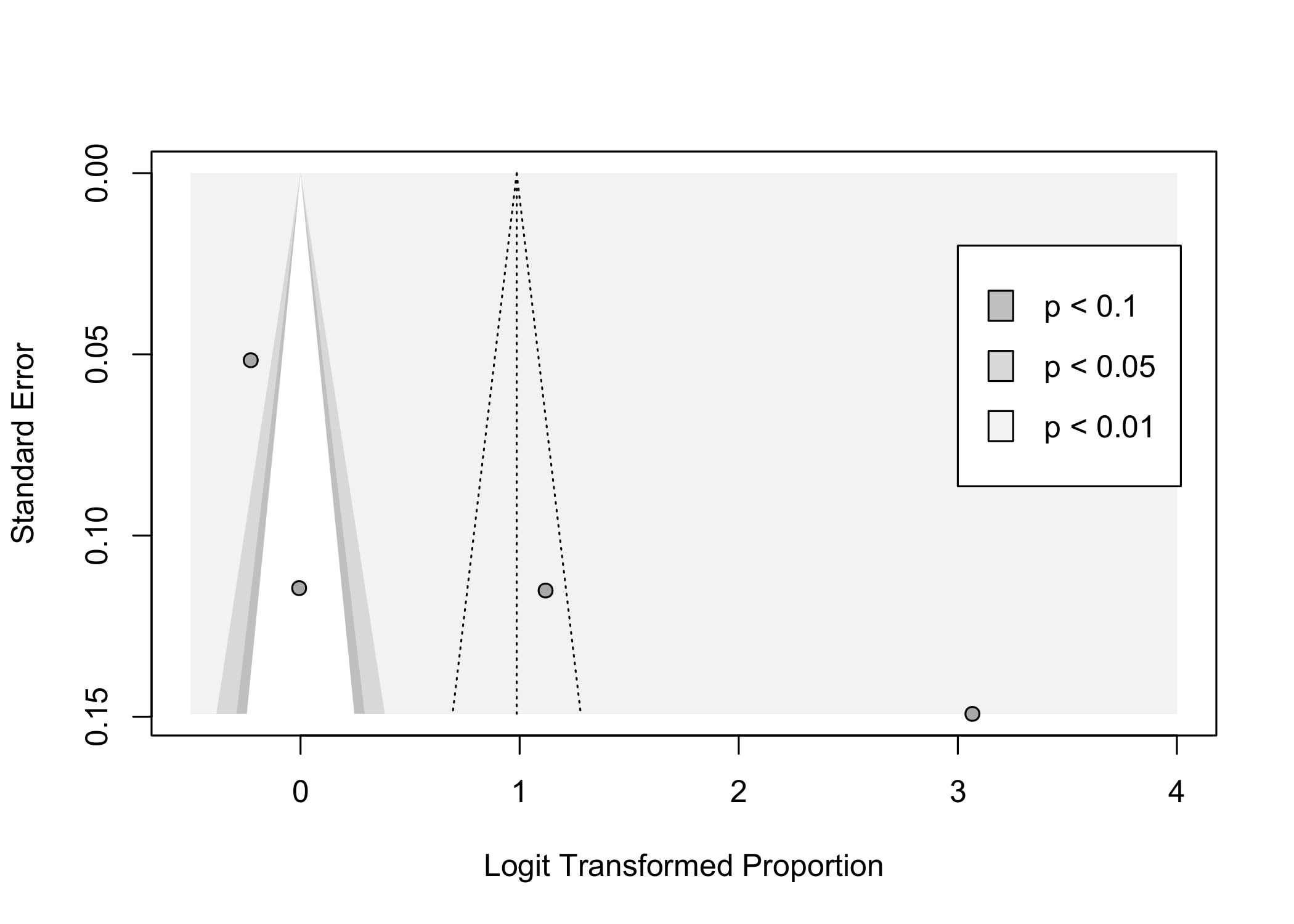
**

**Supplementary Figure 16: Funnel plot of Knowledge of participants about Sore throat as a symptom of Covid-19**

**
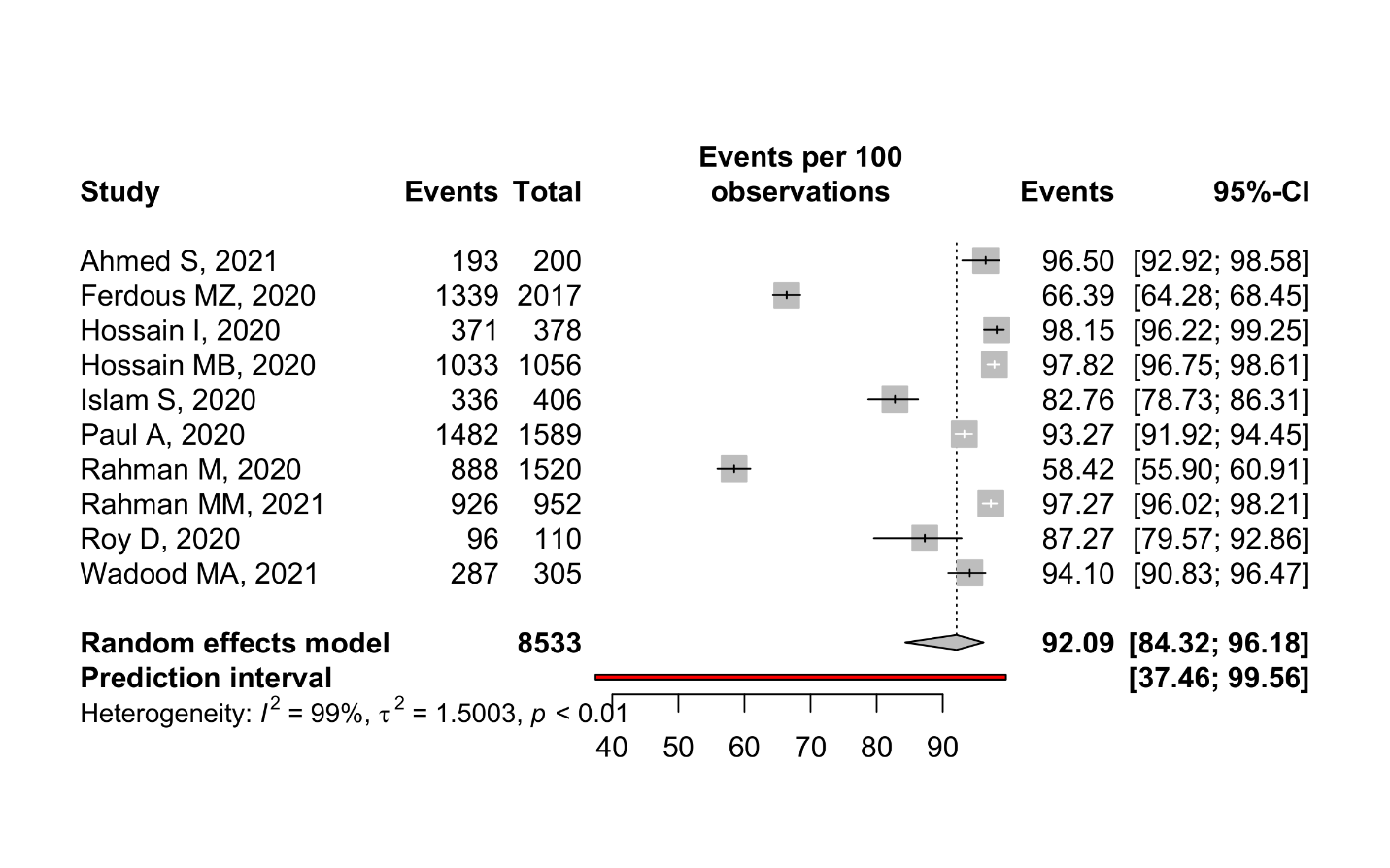
**

**Fig Supplementary Figure 17: Knowledge of participants about transmission of Covid-19**

**
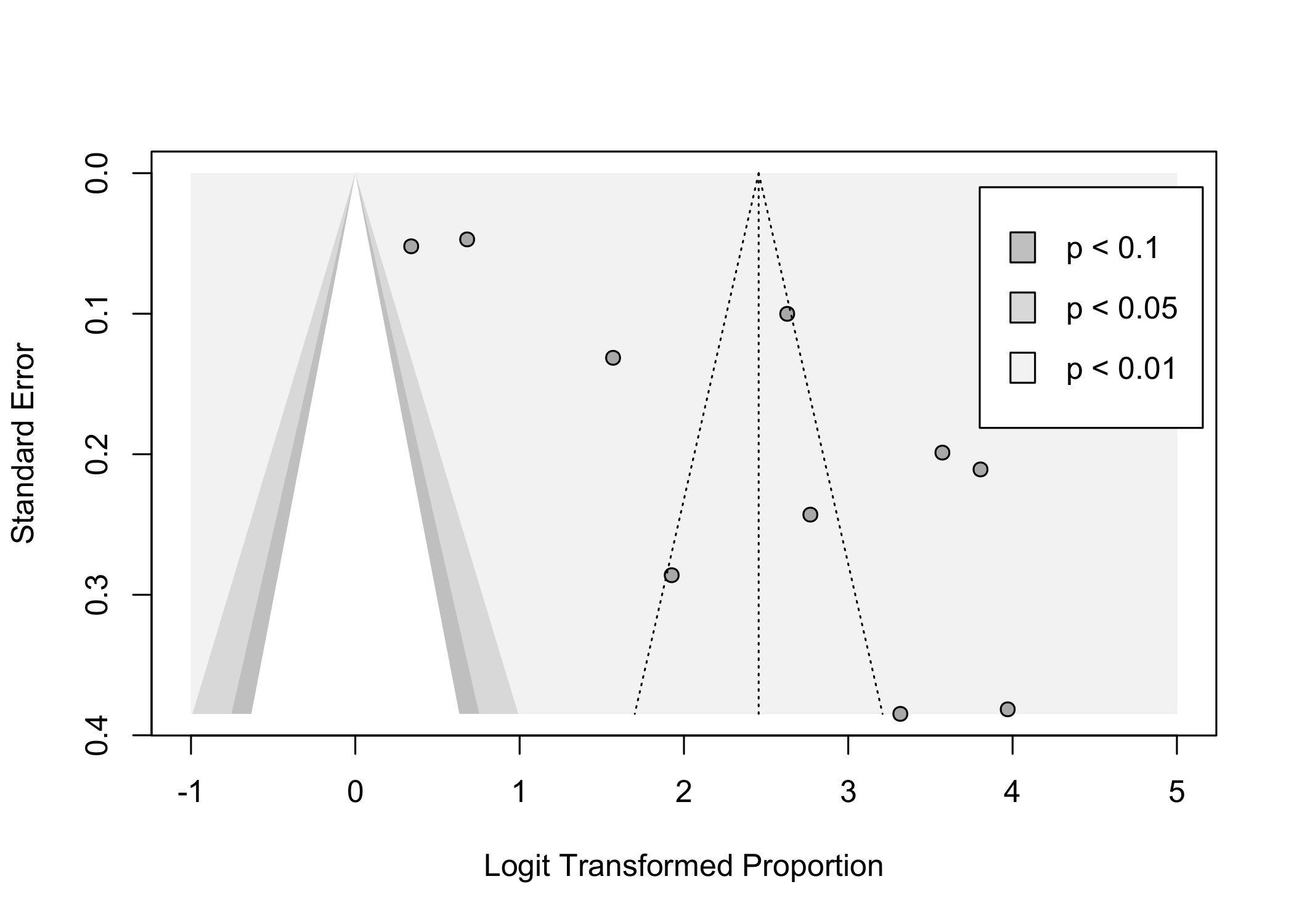
**

**Supplementary Figure 18: Funnel plot of Knowledge of participants about transmission of Covid-19**

**
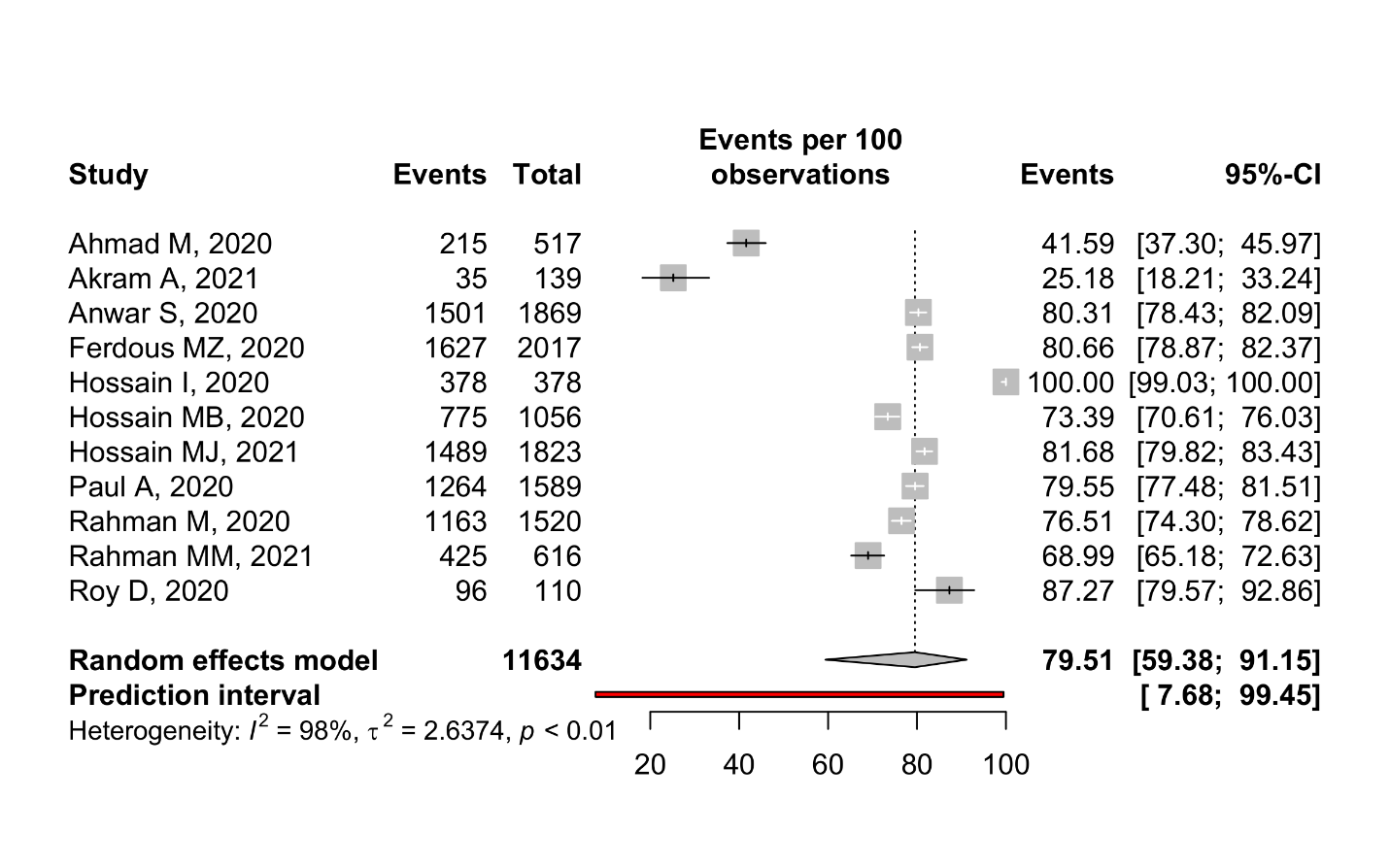
**

**Supplementary Figure 19: Knowledge of participants about Treatments of Covid-19**

**
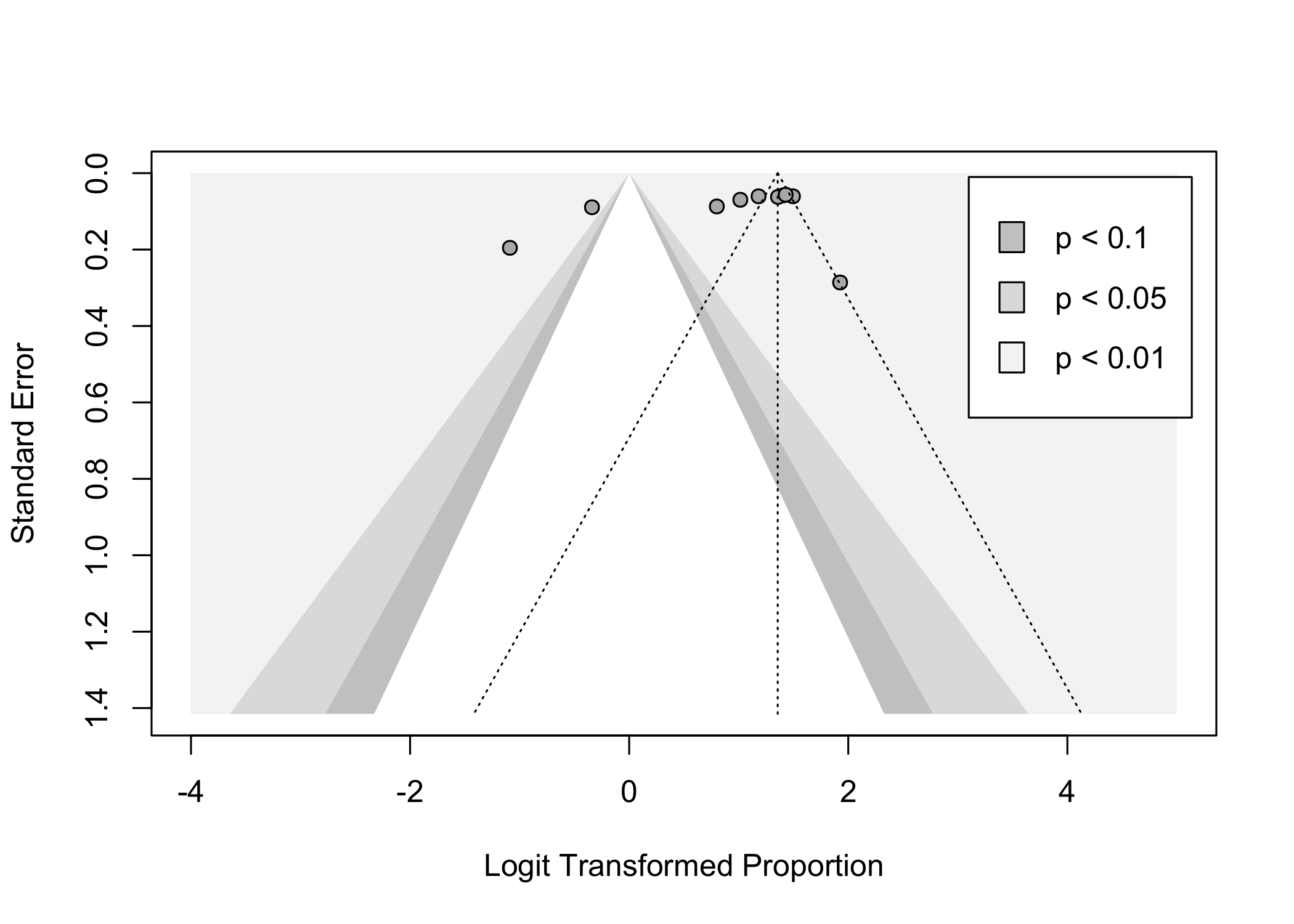
**

**Supplementary Figure 20: Funnel plot of Knowledge of participants about Treatments of Covid-19**

**
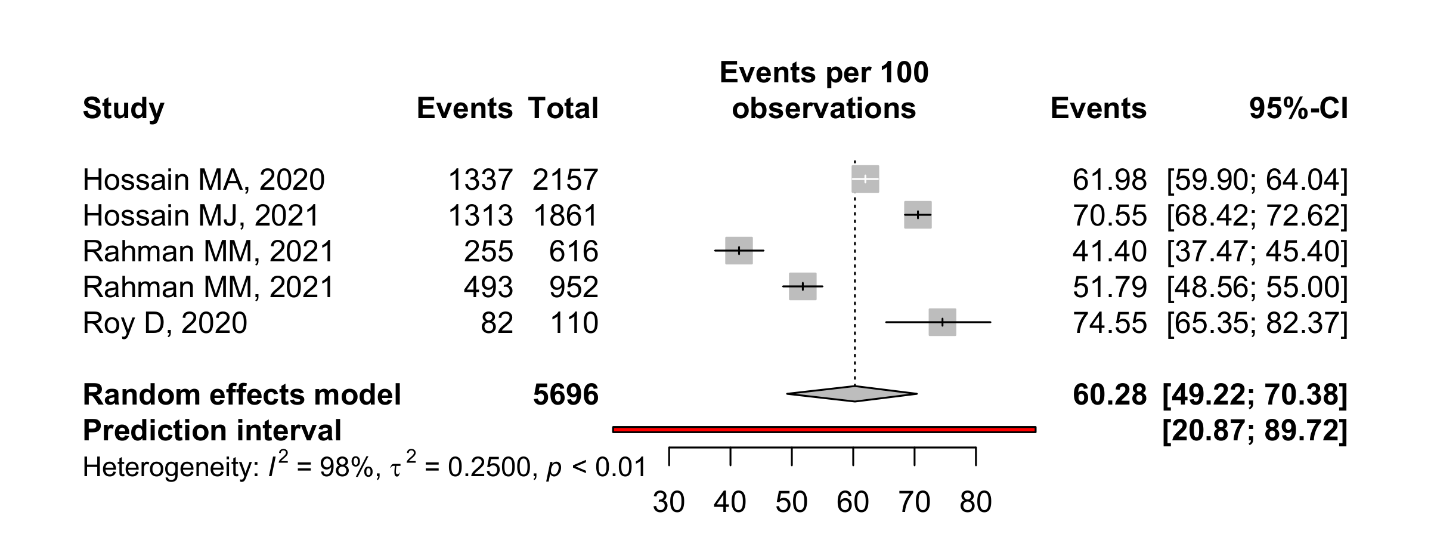
**

**Supplementary Figure 21: Attitude of participants about successful control of covid-19 in Bangladesh**

**
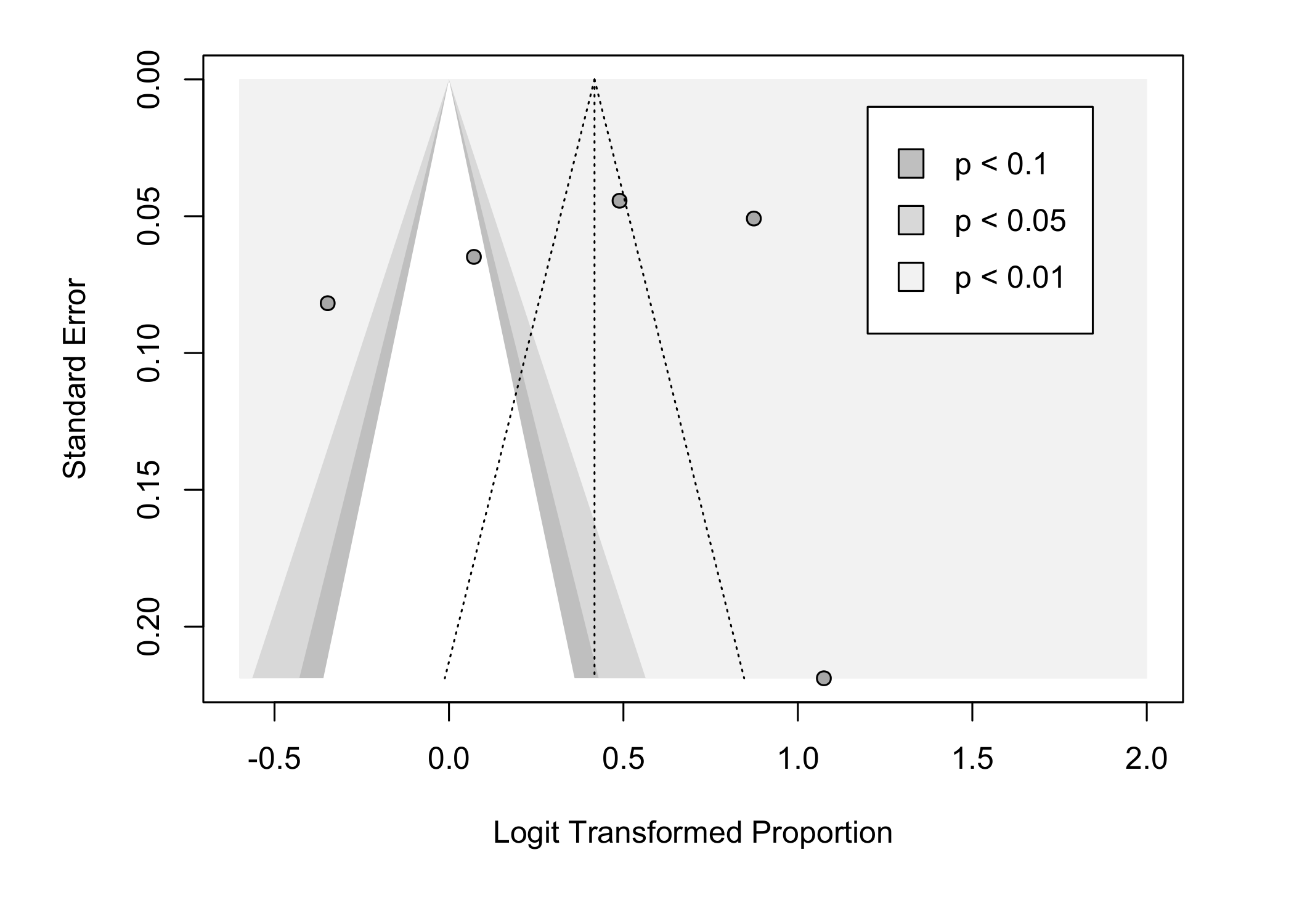
**

**Supplementary Figure 22: Funnel plot of Attitude of participants about successful control of covid-19 in Bangladesh**

**
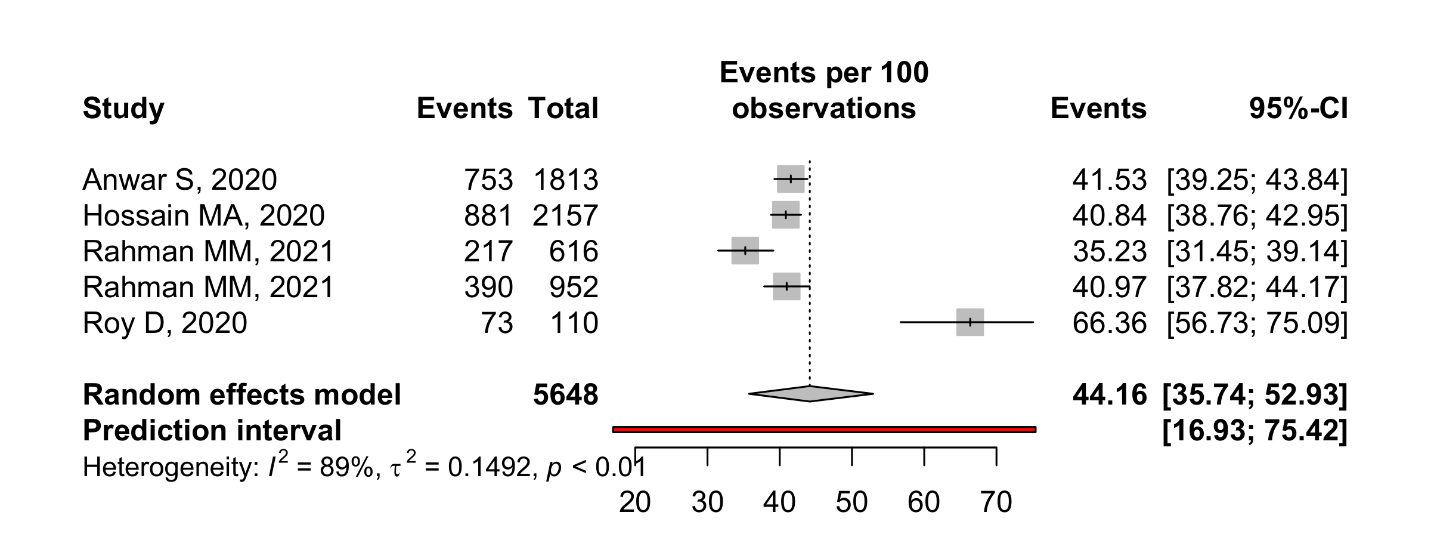
**

**Supplementary Figure 23: Attitude of participants about win of Battle against covid-19 in Bangladesh**

**
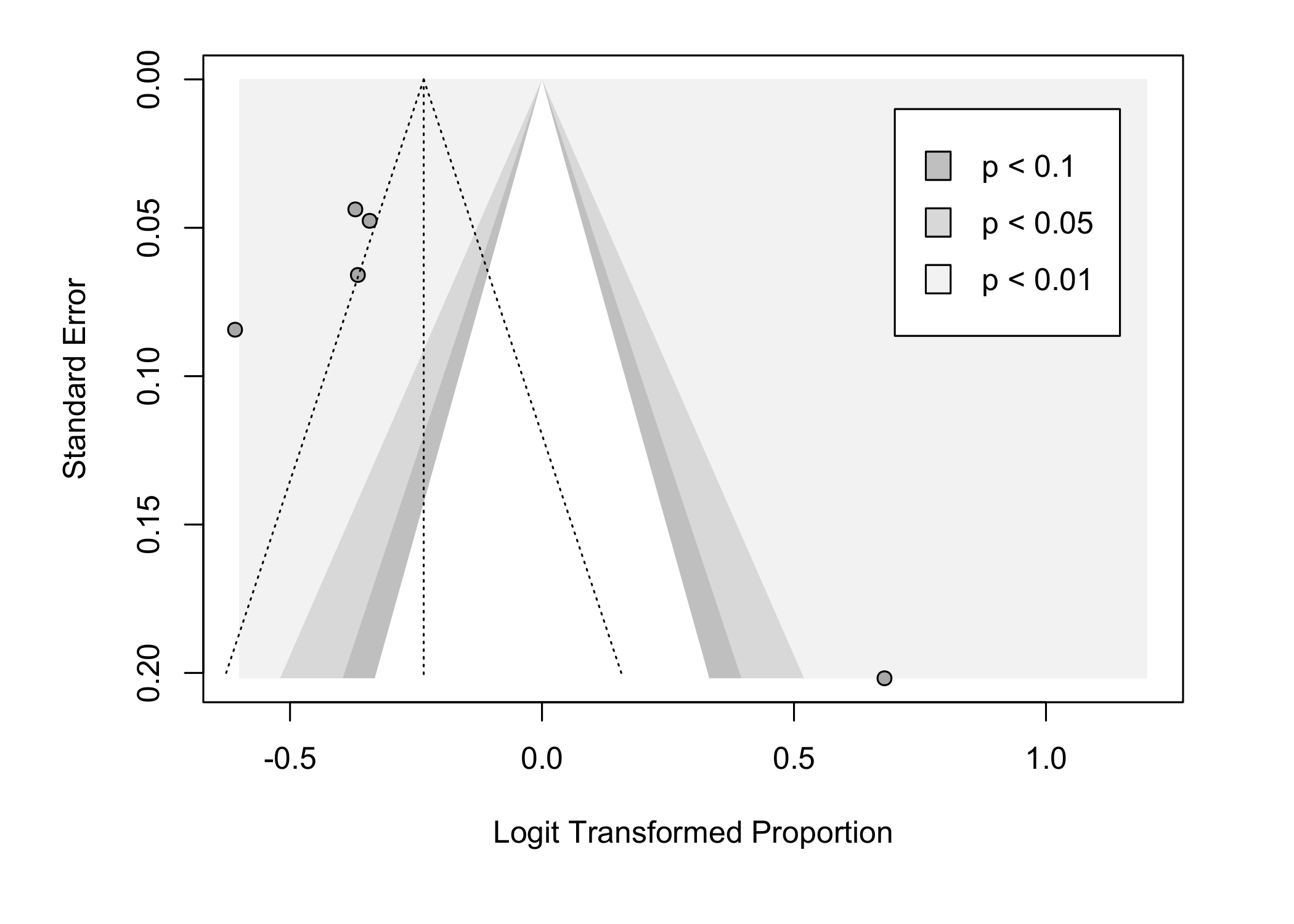
**

**Supplementary Figure 23: Funnel plot of Attitude of participants about win of Battle against covid-19 in Bangladesh**

**
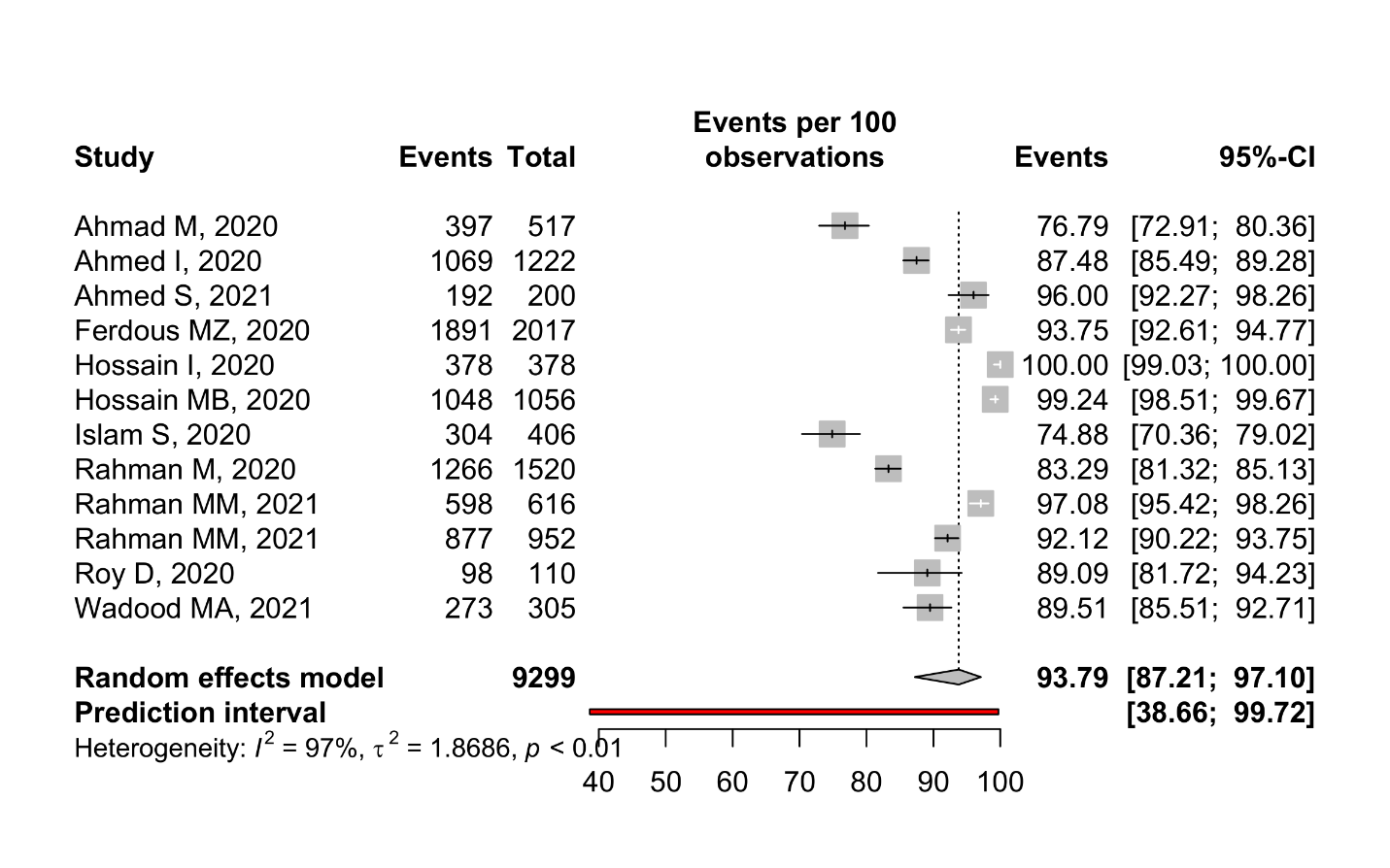
**

**Supplementary Figure 23: Practice level of participants about washing hand regularly**

**
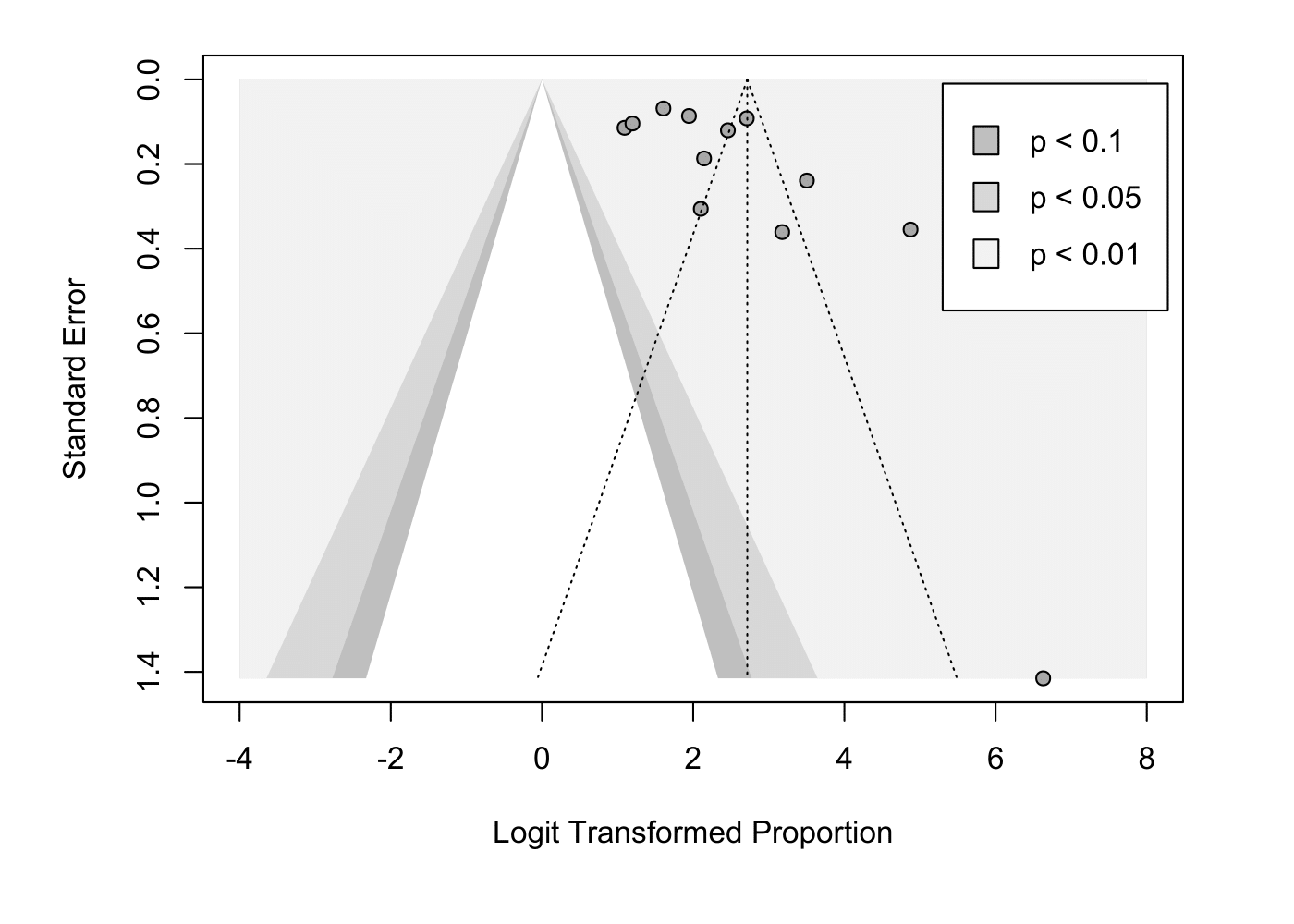
**

**Supplementary Figure 24: Funnel plot of Practice level of participants about washing hand regularly**

**
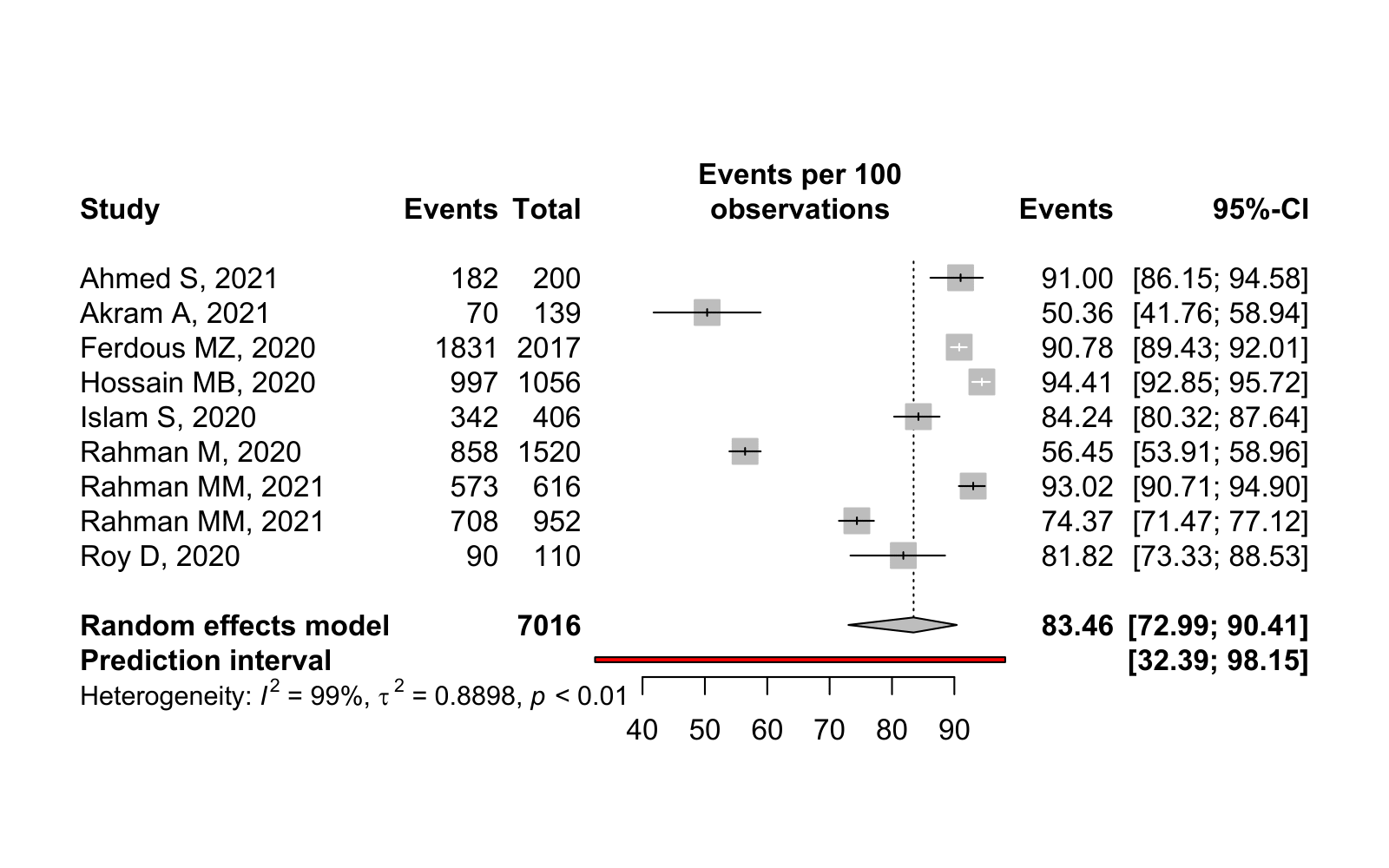
**

**Supplementary Figure 25: Practice level of participants about maintaining Social distance**

**
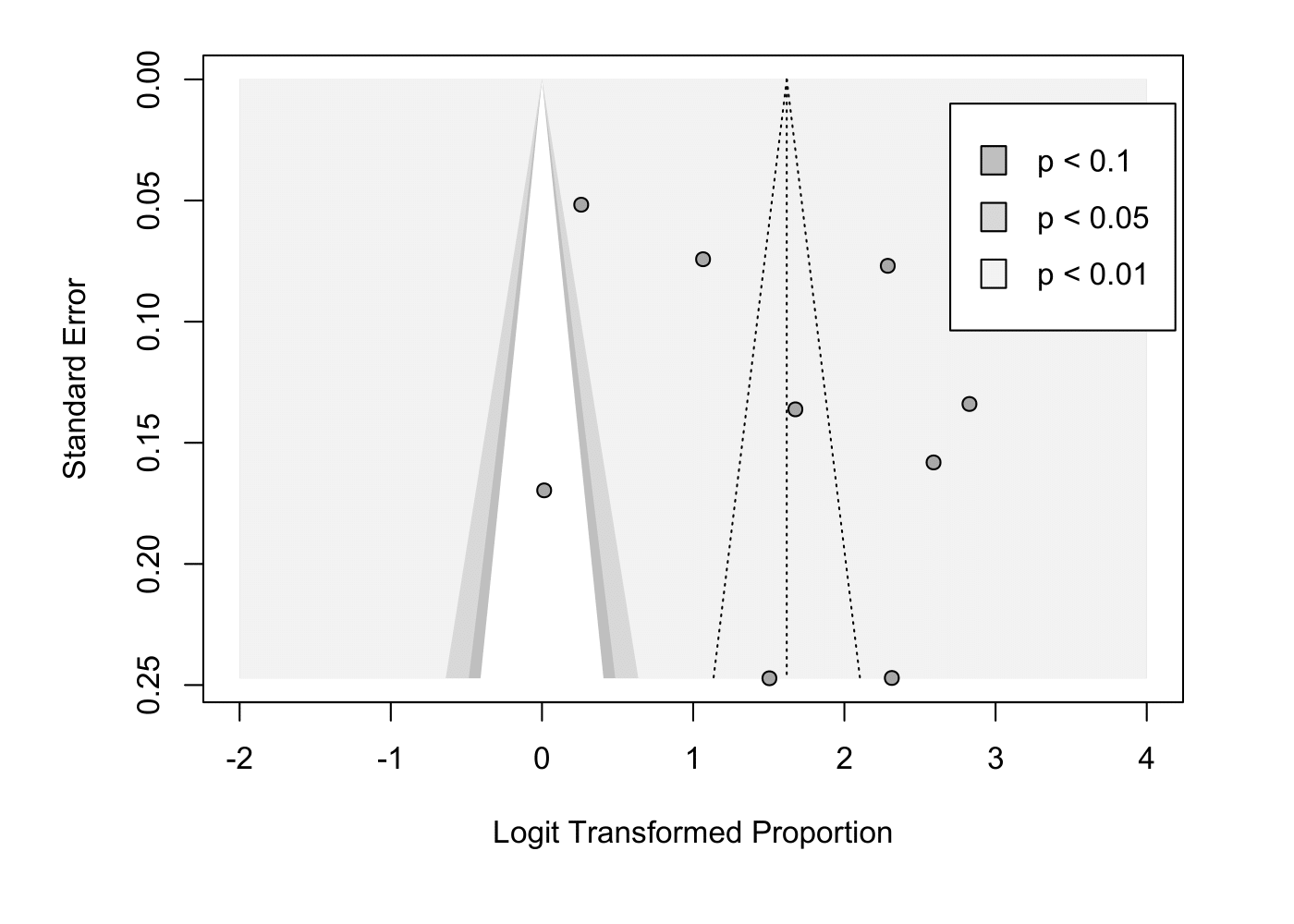
**

**Supplementary Figure 26: Funnel plot of Practice level of participants about maintaining social distance**

**
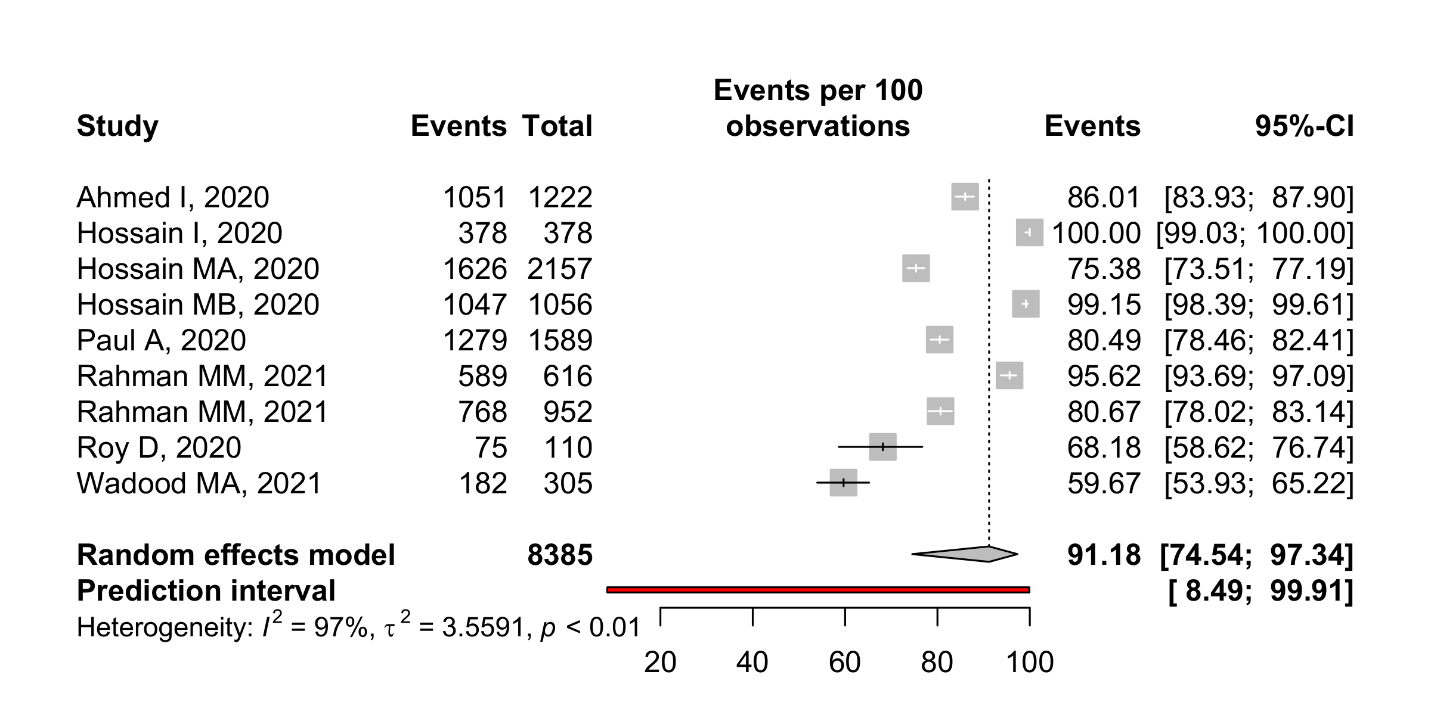
**

**Supplementary Figure 27: Practice level of participants about Avoidance of crowded place**

**
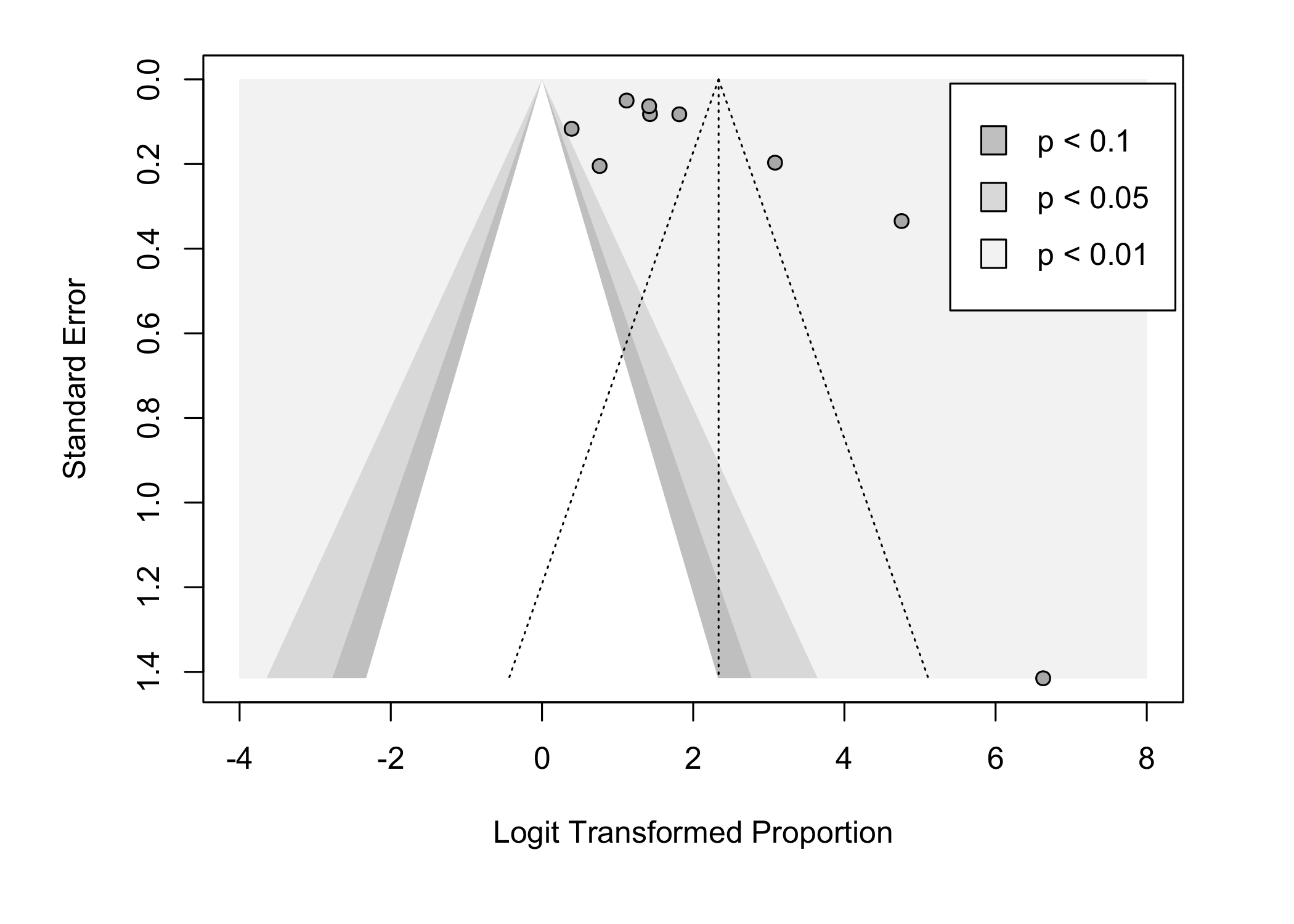
**

**Supplementary Figure 28: Funnel plot of Practice level of participants about Avoidance of crowded place**

**
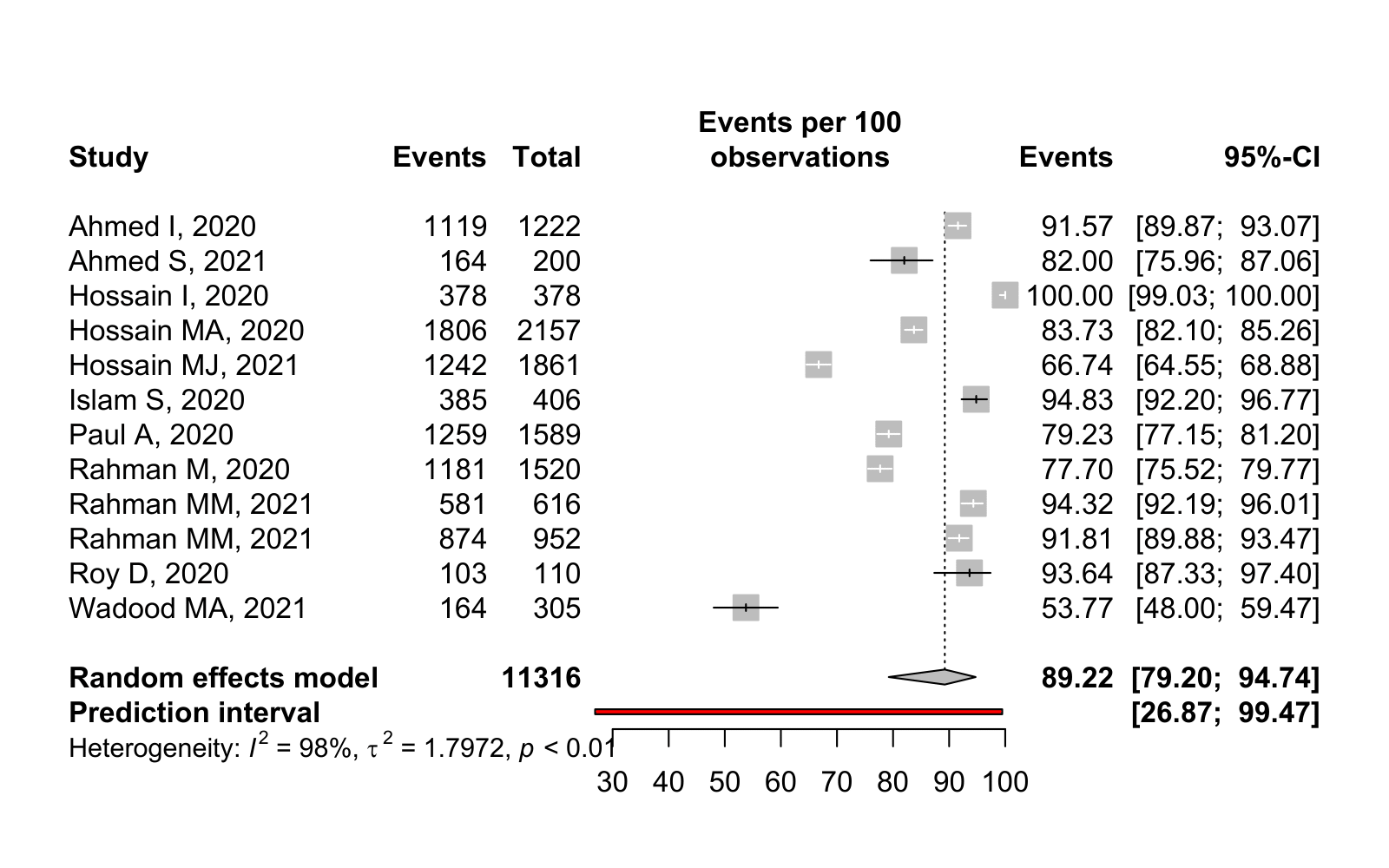
**

**Supplementary Figure 29: Practice level of participants about wearing mask**

**
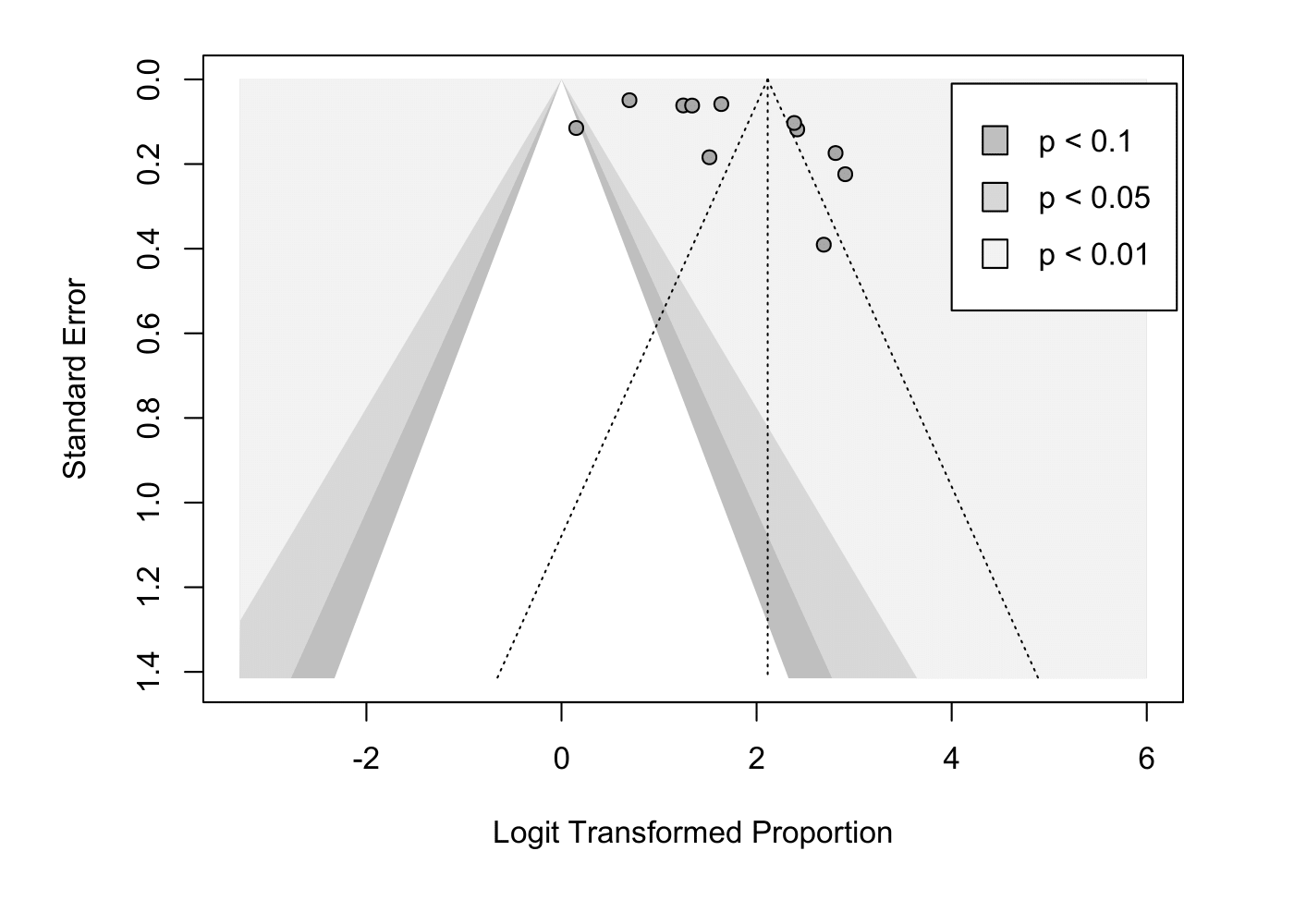
**

**Supplementary Figure 30: Funnel plot of Practice level of participants about wearing mask**
